# Evaluating the Effectiveness of Gamification on Physical Activity: Systematic Review and Meta-analysis of Randomized Controlled Trials

**DOI:** 10.2196/26779

**Published:** 2022-01-04

**Authors:** Alexandre Mazeas, Martine Duclos, Bruno Pereira, Aïna Chalabaev

**Affiliations:** 1 Univ. Grenoble Alpes SENS 38000 Grenoble France; 2 National Research Institute for Agriculture, Food and Environment (INRAE) 63000 Clermont-Ferrand France; 3 Kiplin 44200 Nantes France; 4 Department of Sport Medicine and Functional Exploration University Hospital Clermont-Ferrand, Hospital G. Montpied 63000 Clermont-Ferrand France; 5 Department of Biostatistics unit Clermont-Ferrand University Hospital 63000 Clermont-Ferrand France

**Keywords:** behavior change, eHealth, gamification, health behavior, intervention, meta-analysis, mobile phone, physical activity, systematic review

## Abstract

**Background:**

Gamification refers to the use of game elements in nongame contexts. The use of gamification to change behaviors and promote physical activity (PA) is a promising avenue for tackling the global physical inactivity pandemic and the current prevalence of chronic diseases. However, there is no evidence of the effectiveness of gamified interventions with the existence of mixed results in the literature.

**Objective:**

The aim of this systematic review and meta-analysis is to evaluate the effectiveness of gamified interventions and their health care potential by testing the generalizability and sustainability of their influence on PA and sedentary behavior.

**Methods:**

A total of 5 electronic databases (PubMed, Embase, Scopus, Web of Science, and the Cochrane Central Register of Controlled Trials) were searched for randomized controlled trials published in English from 2010 to 2020. Eligibility criteria were based on the components of the participants, interventions, comparators, and outcomes framework. Studies were included when they used gamified interventions in daily life with an active or inactive control group and when they assessed a PA or sedentary behavior outcome. We conducted meta-analyses using a random-effects model approach. Sensitivity analyses, influence analyses, and publication bias analyses were performed to examine the robustness of our results.

**Results:**

The main meta-analysis performed on 16 studies and 2407 participants revealed a small to medium summary effect of gamified interventions on PA behavior (Hedges *g*=0.42, 95% CI 0.14-0.69). No statistical difference among different subgroups (adults vs adolescents and healthy participants vs adults with chronic diseases) and no interaction effects with moderators such as age, gender, or BMI were found, suggesting good generalizability of gamified interventions to different user populations. The effect was statistically significant when gamified interventions were compared with inactive control groups, such as waiting lists (Hedges *g*=0.58, 95% CI 0.08-1.07), and active control groups that included a nongamified PA intervention (Hedges *g*=0.23, 95% CI 0.05-0.41). This suggests that gamified interventions are not only efficient in changing behavior but also more effective compared with other behavioral interventions. The long-term effect (measured with follow-up averaging 14 weeks after the end of the intervention) was weaker, with a very small to small effect (Hedges *g*=0.15, 95% CI 0.07-0.23).

**Conclusions:**

This meta-analysis confirms that gamified interventions are promising for promoting PA in various populations. Additional analyses revealed that this effect persists after the follow-up period, suggesting that it is not just a novelty effect caused by the playful nature of gamification, and that gamified products appear effective compared with equivalent nongamified PA interventions. Future rigorous trials are required to confirm these findings.

## Introduction

### Background

Physical inactivity and sedentary behavior (SB) are among the leading risk factors for global mortality [1]. Each year, physical inactivity is responsible for >5 million deaths worldwide [2]. In contrast, regular physical activity (PA) prevents the risk of developing chronic diseases [3,4], limits their progression [5,6], and reduces early mortality [7]. In parallel, there is a dose-response relationship between total sedentary time per day and overall mortality [7]. Meta-analyses demonstrate that the risk of mortality in adults increases steadily with a sedentary lifestyle of >3 hours per day and more significantly when this time exceeds 7 hours per day [8]. However, recent studies have suggested that high levels of PA could attenuate or even eliminate the deleterious effects of SB on overall mortality [9].

In this context, it is urgent to develop interventions that can effectively change PA. Therefore, digital health interventions constitute a new opportunity to take care of patients by involving them in their treatment in a dynamic and interactive way. Gamification is a promising avenue to capitalize on the efficacy of digital interventions. Gamification is defined as the use of game design elements in nongame contexts [10]. By integrating game mechanisms in interventions that are initially devoid of them, the purpose of gamification is to integrate into daily life the ingredients that make games enjoyable to motivate participants to engage in PA [11]. The use of *motivational affordances* created by gamification can influence psychological (eg, motivation, attitude, and enjoyment) and physical outcomes (eg, physical capacities) [12] and therefore appears as a potentially powerful technique for behavior change.

By gamifying PA, participants are encouraged to move and walk to play, which tends to make their activities more enjoyable and playful [13]. Unlike serious games, which refer to the use of a full-fledged video game for educational or health purposes (ie, a video game in its entirety as opposed to selected elements or individual features of a game) [10] and require a dedicated time, a location, and implementation [14], gamification techniques are relatively open to varying situational modes of engagement [10] and concern instead global PA in all aspects of daily life (eg, walking, running, or gardening). Gamification is made possible by mobile technologies and wearable devices that can track and collect daily activities in a continuous and web-based manner. This allows for intervening directly on the lifestyle of individuals without adding material or time constraints for the participants.

However, several literature reviews [13,15,16] have reported inconsistent results concerning the use of gamification in behavioral interventions, with some studies demonstrating positive effects and other studies providing more mixed effects. These reviews also emphasized the lack of high-quality studies and highlighted the need for more rigorous trials to isolate the impact of gamification (ie, randomized controlled trials [RCTs]). Importantly, Koivisto and Hamari [15] suggested that the effects of gamification could be smaller when using rigorous experimentation. In sum, these reviews indicate that there is no clear evidence of the effectiveness of gamified interventions. Nevertheless, no meta-analysis has been conducted yet. Quantifying the effect size of gamified interventions and identifying moderators of this effect would provide important information about the effectiveness of such interventions. Moreover, a meta-analysis appears as timely, as there are now enough RCTs to conduct such an analysis.

### This Study

This study is the first to quantify the effects of gamified interventions on PA. Beyond the effect during or just after the intervention, we also seek to evaluate the long-term effects to determine the health relevance of these interventions. Indeed, we reasoned that to be considered effective, gamification must sustain its impacts over the long term and offer more than a short-term novelty effect [11]. The generalizability of gamification to different user populations is also a major issue because it would determine whether gamification can be introduced in health care settings with patients or it is more suited in prevention for healthy audiences.

The objectives of this systematic review and meta-analysis are to answer these research gaps by (1) evaluating the effect of gamified interventions on PA and SB, (2) assessing the long-term or sustained effects of gamified programs, and (3) evaluating the generalizability of gamification across different populations.

## Methods

### Design

This review was conducted according to the PRISMA (Preferred Reporting Items for Systematic Reviews and Meta-Analyses) guidelines [17]. Following recommendations to minimize bias and provide evidence of a priori analysis intentions [18], the study was preregistered under the international prospective register of systematic reviews (PROSPERO; registration number: CRD42020186882) and on the Open Science Framework (OSF) [19]. Moreover, all materials and data are available on the OSF page of the project to facilitate reproducibility and transparency of this review [20].

### Search Strategy and Information Sources

We conducted a systematic literature search using five electronic databases: PubMed, Embase, Scopus, Web of Science, and the Cochrane Central Register of Controlled Trials. We combined alternate terms and keywords representing synonyms for the outcomes (PA or SB), intervention (gamification), device (eHealth), and trial (see Table S1 in Multimedia Appendix 1 [21-36] for an overview of the terms used). The search strategy was reviewed by an academic librarian. All databases were searched individually from January 1, 2010 (2010 being the date of the widespread adoption of the term *gamification* [10]), to December 31, 2020, and the research was restricted to English-language texts. The complete search equations for all databases are available in Multimedia Appendix 1. In addition, we complemented our search with reference harvesting from the included studies and overview articles.

### Eligibility Criteria

Studies were eligible for inclusion if they were RCTs and if they met other criteria based on the participants, interventions, comparators, and outcomes framework.

#### Participants

This review focused on the general population regardless of age, gender, or health status (ie, patients with chronic diseases were also included). We excluded studies involving participants with contraindications to PA or with diseases preventing them from engaging in PA or understanding the principles of the game (intellectual and cognitive impairments).

#### Intervention

Digital interventions targeting PA or SB and incorporating game elements and gamification techniques, such as points, levels, rewards, leaderboards, narratives, and teams, were of interest. We clearly distinguished between gamification and related constructs, such as serious games. Therefore, we excluded interventions based on active video games (ie, electronic games that allow players to physically play with the images on screen) that are more comparable with serious games than with gamified products.

#### Comparators

Studies that attempted to compare gamified interventions with control groups without gamification elements in a randomized design were integrated in the review. These groups could be either inactive (nonexposed control group, such as a waiting list) or active (another nongamified intervention).

#### Outcomes

In this review, we included studies assessing change in total PA or leisure PA (quantity in metabolic equivalent of task [MET] hour per week or MET minute per week or in duration, energy expenditure [METs], moderate to vigorous PA [MVPA], step count, walking time, and active minutes) and change in time spent in SB (total time, leisure time, work, time spent in front of the computer, and time spent in front of television). These outcomes were continuous data either objectively measured (through accelerometers, pedometers, and smartphones) or subjectively measured by self-reported questionnaires. Data measured objectively were always prioritized in the analyses over self-reported questionnaires, which are more susceptible to bias with a potential overestimation of PA [37].

In addition, studies were excluded if they came from a review, commentary, or conference abstract; if they included data previously published in another study; if they were not randomized and controlled; if they were not written in English; and if they were published before 2010.

### Screening

In total, 2 authors (AM and AC) independently screened the titles and abstracts resulting from the search. Full texts of the potential included studies were checked before inclusion. Disagreements were resolved by discussion or by consulting a third author (MD), and agreement was measured using the *κ* statistic. A complete list of excluded studies is available on the OSF page of the project.

### Data Extraction

In the data collection process, AM and AC extracted data independently and were blinded to each other using a predetermined and tested template. Disagreements were resolved by discussion or consultation with a third author (MD). Extracted data included the results of each study and three types of potential moderators:

1. Population-level moderators to assess the generalizability of the intervention (population characteristics, age, gender, and pathology).
2. Intervention-level moderators to better understand gamification mechanisms (theoretical model used to develop the intervention, gamification elements, and modality of the intervention [eg, internet-based, smartphone app, and presence of social incentives]).
3. Outcome-level moderators (outcomes, measure of PA or SB, and device).

### Risk of Bias Assessment

For each eligible study, 2 reviewers (AM and AC) assessed the risk of bias using the purpose-built Cochrane risk of bias tool (Table 8.5 in the Cochrane Handbook for Systematic Reviews of Interventions [38]), which evaluates 7 domains (sequence generation, allocation concealment, blinding of participants and personnel, blinding of outcome assessment, incomplete outcome data, selective outcome reporting, and other risk of bias). A judgment of the potential risk of bias was made from the extracted information and rated as *high risk*, *low risk*, or *unclear* if the related information was not available. These evaluations of bias are reported in the review and included in the analysis, and a measure of agreement with the *κ* statistic was calculated. After the full assessment, we decided not to present the item *blinding of participants and personnel* in the review because it was similar for all studies, which were rated as *high risk*, blinding being unfeasible for this kind of intervention.

### Data Synthesis and Statistical Analyses

First, data were synthesized in a qualitative review assessing the key elements of the studies and highlighting intervention differences. This qualitative review integrates all studies that met the eligibility criteria, including those for which we were unable to extract the data.

For the quantitative analysis, means and SDs of continuous PA or SB outcomes from individual studies were compiled when available or estimated using the method by Hozo et al [39] when median and IQR were reported. When the necessary data were not available in the original article, we first requested them from the authors. If data could not be obtained, we extracted them from the graphs when available. If this was not possible, we excluded the study from the quantitative analysis.

A global meta-analysis was conducted to obtain a summary effect. In addition, when sufficient data were available (ie, 4 studies or more reporting an outcome), we conducted different meta-analyses for each specific outcome (steps, MVPA, and time in SB) and for the follow-up effect. To address the nonindependence of data caused by study effect, random-effects models [40] were preferred to the usual statistical tests. In addition, the Hartung–Knapp–Sidik–Jonkman method was used to reduce the production of false positives inherent to the DerSimonian–Laird method [41] and to obtain more robust estimates of variance. Continuous outcomes were analyzed using standardized mean difference (SMD) to account for different measurement instruments or mean difference (MD) when the measurements were close enough. We computed Hedges *g* [42] for effect sizes, which is similar to Cohen *d* but corrects for small sample bias, which are recurrent in the studies included. Thus, a Hedges *g* of 0.2 represents a small effect; 0.5, a moderate effect; and 0.8, a large effect [43]. We computed SMDs for outcome scores after the intervention (presented in the review) and change-from-baseline outcomes. Scores on postintervention effect sizes refer to treatment group results compared with the control group results after interventions. Change-from-baseline score effect sizes were calculated as a comparison between the treatment group pre–post effect size and control group pre–post effect size.

For studies that included multiple outcomes, we kept in the main analysis the primary outcome targeted in the initial article. If none of the PA outcomes reported by a study were the primary ones, we selected the one that was the most relevant from the perspective of the intervention and the original experiment. In designs with multiple time measurements, the assessment that was the most proximal to the end of the intervention was conserved. A time assessment had to be performed >2 weeks after the end of the intervention to be included in the follow-up analysis. When studies included multiple intervention groups with gamification features, they were combined into one group following the formulae recommended by the Cochrane Handbook [38]. Studies including multiple control groups could be integrated into different subgroup analyses if they compared their gamified intervention to both an active and an inactive control group.

Statistical heterogeneity was tested using forest plots and the *I^2^* statistic, which is the most common metric for measuring the magnitude of between-study heterogeneity and is easily interpretable (0%-40% might not be important, 30%-60% may represent moderate heterogeneity, 50%-90% may represent substantial heterogeneity, and 75%-100% may represent considerable heterogeneity) [44]. We conducted different influential analyses to address between-study heterogeneity. We first explored the presence of outliers, defined as studies with CIs that do not overlap with the CI of the summary effect. We also performed leave-one-out analyses, which recalculated the summary effect several times, with 1 study omitted each time. Finally, we performed a Baujat plot [45], which is a diagnostic plot to detect sources of heterogeneity in the meta-analysis by comparing the contribution of each trial in the pooled effect with the overall Cochran *Q* test for heterogeneity.

We applied different methods to detect publication bias (funnel plot, Egger regression test [46], and Duval and Tweedie trim-and-fill procedure [47]). In addition, another approach to determine the evidential value of studies included in the analysis is to check the statistical power of individual studies. Therefore, we performed a sunset funnel plot [48], which is a funnel plot variant that visualizes the statistical power of each study included in the meta-analysis based on the summary effect size.

Thus, sensitivity analyses were conducted to address studies with a high risk of bias or a strong heterogeneity in the sample or studies identified as outliers. Subgroup analyses were conducted to explore possible sources of heterogeneity and test for population differences. Therefore, we conducted tests for subgroup differences using a random-effects model. In addition, moderation analyses were performed to explore the impact of potential explanatory variables and moderators on the effect size with meta-regressions when sufficient data were available (ie, at least 10 studies for each explanatory variable [38]). The results were expressed as regression coefficient estimates, 95% CIs, and *P* values.

For crossover trials, we first checked whether carry-over or period effects were problematic in the original texts of studies. For cluster randomized trials, we checked if the influence of the different clusters was not too important, analyzing the values of the intraclass correlation coefficient in the studies. Then, in the absence of sufficient information in the published articles, we addressed these studies as traditional parallel trials. Nevertheless, this procedure increased the probability of a unit of analysis error. Therefore, we performed sensitivity analyses without clusters and crossover trials to test the robustness of our results.

A summary of the analytical procedure is available in Multimedia Appendix 1 (Figure S1). Analyses were performed on R (The R Project for Statistical Computing) using the *dmetar* package [49]. Risk of bias summary and risk of bias graphs were made via the *robvis* R package [50].

## Results

### Characteristics of Included Studies

**Figure 1:**
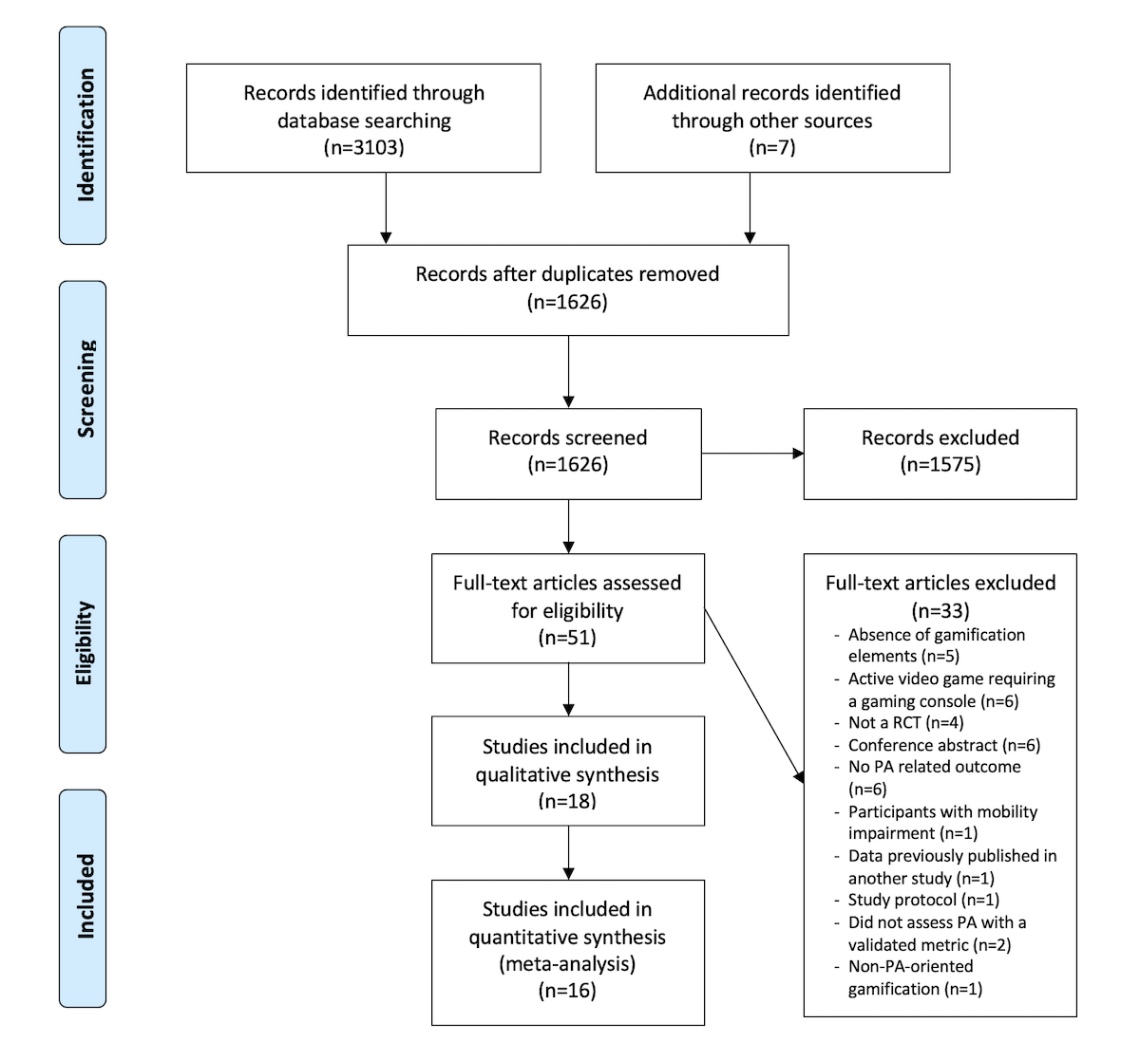
PRISMA (Preferred Reporting Items for Systematic Reviews and Meta-Analysis) flowchart of the literature search and screening process. PA: physical activity; RCT: randomized controlled trial.

We screened the titles and abstracts of 1626 articles, and 51 full-text articles were assessed for eligibility according to the inclusion criteria. Finally, 18 articles [21-36,51,52] were included in the qualitative analysis and 16 were included in the meta-analysis (Figure 1). The *κ* value of agreement for the screening phase was 0.64 between the 2 authors, reflecting good agreement [53].

Table 1 provides an overview of the characteristics of the included studies. The 16 studies included in the quantitative analysis involved 2407 participants aged 9-73 years (mean 35.7 years, SD 17.2 years), with sample sizes ranging from 20 to 602. Overall, 67% (12/18) of studies included adult participants [22,24-30,32,34-36] and 33% (6/18) of studies included adolescents (ie, <18 years [21,23,31,33,51,52]). A total of 22% (4/18) of studies included patients with chronic diseases (ie, obesity [27,36], type 2 diabetes [35], and cardiovascular disease [28]).

A total of 6 trials were conducted in Europe; 4 in the United States; 3 in Australia and New Zealand; 3 in Canada; and 2 in Asia. Studies were published between 2014 and 2020, with 39% (7/18) published after 2018.

Most studies were based on a smartphone app (n=10; [23,28,30-35,51,52]), web-based (n=3; [21,24,29]), or both (n=4; [22,26,27,36]). Nishiwaki et al [25] used a pedometer with computerized game functions. The duration of the intervention varied from 1 to 24 weeks, with a mean of 11.8 weeks, and the most common length was 24 weeks. The most used game mechanics were internet-based rewards, such as badges, medals, or trophies (13/18, 72%; [21,22,24,26-28,30,32,33,35,36,51,52]), teams or leagues (13/18, 72%; [21-24,26,27,29,32-34,36,51,52]), levels (9/18, 50%; [22,26,27,29,33,35,36,51,52]), points or scores (7/18, 39%; [22,26,27,29,30,35,36]), or the presence of a leaderboard (7/18, 39%; [22,29,30,33,34,51,52]). Almost all studies included social incentives such as team collaboration, social networking, and messaging facilities in their intervention (15/18, 83%; all except Nishiwaki et al [25], Direito et al [31], and Höchsmann et al [35]).

**Table 1:** Characteristics of included studies.

Study	Participants	Intervention	Theory	PA^a^ outcomes
Corepal et al [21]^b^	Adolescents, n=224 (aged 12-14 years; 47% male participants)	The “StepSmart Challenge” was a web-based intervention that used gamification strategies to encourage and support PA behavior change; duration: 22 weeks	SDT^c^	Daily step count and MVPA^d^ (min/day) objectively measured (Actigraph GT3x)
Dadaczynski et al [22]^b^	Adult workers in an automobile manufacture, n=144 (65% male participants)	“Healingo Fit” had the objective to promote low levels of PA using a tracking-based approach measuring PA with a Fitbit pedometer and a gamified intervention accessible by desktop and mobile devices; duration: 6 weeks	SCT^e^, TPB^f^, and health action process approach	Self-reported VPA^g^, MPA^h^, and minutes walked (min/week; IPAQ^i^)
Direito et al [31]^b^	Adolescents, n=35 (mean age 15.7 years, SD 1.2 years; 45% male participants; BMI 22.85)	“Zombies, run! 5K Training app” was a fully automated training program designed to improve fitness, combined with an immersing and fun story; duration: 8 weeks	SDT	MVPA, VPA, MPA, and LPA^j^ and sedentary time (min/day) objectively measured (Actigraph GT3x) and self-reported PA (PAQ-A^k^)
Edney et al [32]^b^	Adults, n=284 (mean age 41.2 years, SD 11.2 years; 25% male participants; BMI 30.1)	“Active Team” was a mobile app designed to encourage inactive adults to meet PA guidelines. Gamification and social features were implemented to increase the social comparison, support, and influence among participants; duration: 12 weeks	SCT	MVPA (min/day) objectively measured (GENEActiv) and self-reported PA (Active Australia Survey)
Garde et al [51]	Adolescents, n=47 (mean age 10.3 years, SD 1.9 years; 34% male participants; BMI *z-*score 0.35)	“MobileKids Monster Manor” was a mobile exergame synchronized with an external activity monitor. The overall goal was to complete the story with PA and steps; duration: 1 week	SDT	Daily step count and active min/day objectively measured (Tractivity)
Garde et al [33]^b^	Adolescents, n=56 (mean age 11.3 years, SD 1.2 years; 62% male participants; BMI *z-*score 0.28)	“MobileKids Monster Manor” was a mobile exergame synchronized with an external activity monitor. The overall goal was to complete the story with PA and steps; duration: 1 week	SDT	Daily step count and active min/day objectively measured (Tractivity)
Garde et al [52]	Adolescents, n=37 (mean age 10.6 years, SD 0.5 years; 43% male participants; BMI *z-*score 0.21)	“MobileKids Monster Manor” was a mobile exergame synchronized with an external activity monitor. The overall goal was to complete the story with PA and steps; duration: 2 weeks	SDT	Daily step count and active min/day objectively measured (Tractivity)
Gremaud et al [34]^b^	Adult office workers, n=144 (mean age 40.5 years, SD 11.4 years; 76% male participants; BMI 29.7)	“MapTrek” was a mobile health platform that gamified Fitbit use for promoting PA by placing users in a series of internet-based walking races; duration: 10 weeks	SCT	Daily step count and daily active minutes count objectively measured (Fitbit Zip activity monitor)
Höchsmann et al [35]^b^	Patients with type 2 diabetes mellitus and obesity, n=35 (mean age 58.5 years; 53% male participants; BMI 32)	The intervention was a mobile app including a storyline, virtual rewards, individualized exercises, and daily PA promotion through a game; duration: 24 weeks	Taxonomy of behavior change techniques	Daily step count objectively measured (Garmin Vivofit 2)
Kurtzman et al [36]^b^	Adults with obesity, n=196 (mean age 41.4 years, SD 12.2 years; 13% male participants; BMI 36.2)	Participants were in teams of 2 and had to complete weekly goal targets to win points and badges; duration: 24 weeks	Behavioral economics	Mean step count objectively measured (Withings wrist-worn device)
Leinonen et al [23]^b^	Adolescents, n=496 (mean age 17.8 years, SD 0.6 years; 100% male participants; BMI 23.1)	The intervention was an app proposing a mixed-reality conquering game in which physical and social activities are rewarded; duration: 24 weeks	Transtheoretical model	Daily MVPA and daily sedentary time objectively measured (Polar Active)
Maher et al [24]^b^	Adults, n=110 (mean age 35.6 years, SD 12.4 years; 42% male participants)	“Active Team” was a Facebook (Meta Platforms) app designed to encourage inactive adults to meet PA guidelines. Gamification and social features were implemented to increase the social comparison, support, and influence among participants; duration: 8 weeks	TPB	Self-reported MVPA, VPA, MPA, and minutes walked (min/week; Active Australia Survey)
Nishiwaki et al [25]^b^	Adults, n=20 (mean age 31 years, SD 3 years; 30% male participants; BMI 21.5)	Participants wore an activity monitor with computerized game functions, such as a story, a character, and objectives; duration: 6 weeks	—^l^	Daily step count and MVPA (metabolic equivalent of tasks hour/day) objectively measured (Lifecorder EX)
Patel et al [26]^b^	Adults, n=200 (mean age 55.9 years, SD 9.9 years; 44% male participants; BMI 27.1)	Participants were entered into a game with their family in teams and had to complete weekly goal targets to win points and badges; duration: 12 weeks	Behavioral economics	Daily step count objectively measured (Withings wrist-worn device)
Patel et al [27]^b^	Adults with overweight and obesity, n=602 (mean age 38.7 years, SD 10.4 years; 69% male participants; BMI 29.6)	Participants had to complete weekly goal targets to win points and levels. There were 3 versions of the intervention: support, collaboration, and competition; duration: 24 weeks	Behavioral economics	Daily step count objectively measured (Withings wrist-worn device)
Paul et al [28]^b^	Patients who survived stroke, n=23 (mean age 55.8 years, SD 10.7 years; 48% male participants; BMI 24.5)	In the “STARFISH” app, participants had to complete their PA objectives to improve their avatar; duration: 6 weeks	Control theory and Michie taxonomy of behavior change	Daily step count, sedentary time, and walking time (min/week) objectively measured (ActivPAL)
Thorsteinsen et al [29]^b^	Adults, n=21 (mean age 55.3 years, SD 11.2 years; 52% male participants)	The intervention “Lifestyle Tool” consisted of a rule-based website designed to help people plan and monitor their PA. The tool incorporated social and individual gaming components to increase motivation and engagement; duration: 12 weeks	—	Self-reported weekly activity minutes (daily report form)
Zuckerman and Gal-Oz [30]^b^	Students, n=59 (mean age 23.4 years, SD 1.4 years; 25% male participants)	“StepByStep” was an accelerometer-based mobile app with virtual rewards and social comparison intended to motivate people to incorporate more walking into their daily routine; duration: 1.5 week	SDT	Walking time (min/day) objectively measured (smartphone accelerometer)

^a^PA: physical activity.

^b^The studies included in the meta-analysis.

^c^SDT: self-determination theory.

^d^MVPA: moderate to vigorous physical activity.

^e^SCT: sociocognitive theory.

^f^TPB: theory of planned behavior.

^g^VPA: vigorous physical activity.

^h^MPA: moderate physical activity.

^i^IPAQ: International Physical Activity Questionnaire.

^j^LPA: light physical activity.

^k^PAQ-A: Physical Activity Questionnaire for Adolescents.

^l^No theory mentioned.

Studies comparing gamified interventions with active control groups used a similar intervention without game elements (ie, an equivalent nongamified app [30-32] or a self-monitoring intervention with wearables or activity monitors [25-27,34,36]).

In most studies, the interventions were based on theoretical models. A total of 6 studies [21,30,31,33,51,52] were based on the self-determination theory [54]; 5 [22-24,32,34] on sociocognitive models (ie, the transtheoretical model [55], the social cognitive theory [56], the theory of planned behavior [57], and the health action process approach [58]); and 3 [26,27,36] on behavioral economics models.

Outcomes measured in trials were diverse: they used either total PA duration or MVPA duration, SB duration, daily step count, walking duration, or active minute count. A total of 13 experiments measured PA objectively using devices such as triaxial accelerometers (n=7; [21,28,31-33,51,52]), wearable devices for the general population (ie, Fitbit, Garmin, Polar, and Withings monitors; n=6; [23,26,27,34-36]), pedometers (n=1; [25]), or smartphones (n=1; [30]), and 5 assessed PA with self-reported measures (International Physical Activity Questionnaire [22], Physical Activity Questionnaire for Adolescents [31], Active Australian Survey [24,32], or other [29]). A total of 6 trials [21,24,26,27,32,36] completed a follow-up assessment from 12 to 30 weeks (mean 14.4 weeks) after the end of the intervention.

A total of 2 studies [51,52] were excluded from the meta-analysis and were only integrated in the qualitative review because we were unable to extract their results.

### Risk of Bias

The 2 authors extracted the risk of bias data with a *κ* coefficient of 0.79, which is synonymous with excellent agreement [53]. Multimedia Appendices 2 [21-36] and 3 present the authors’ judgments about each risk of bias item presented as percentages across all included studies in the meta-analysis and an overview of the different biases for each study. Overall, 1 study [28] was rated as high risk for sequence generation because assignments were based on recruitment order. Therefore, this study was also at a high risk for allocation concealment. A total of 3 studies [24,29,30] were at high risk of bias for the *blinding of outcome assessment* item because they measured PA using only self-reports. In total, 5 studies [23,29,30,49,52] were at high risk of bias for the *incomplete outcome data* item because they reported high dropout rates and did not include intention-to-threat analyses and 5 studies [25,28,29,31,52] were rated at unclear risk for the *selective outcome reporting* item because they had not been preregistered or published in a protocol-study. Finally, 2 studies had other high risks of bias. The first one [25] was a crossover trial conducted without a washout condition, and the other one [21] was a cluster randomized trial with no control of clustering, no consideration of the clustering in the statistical analysis, and no test of baseline differences among groups.

### Summary Effect

**Figure 2:**
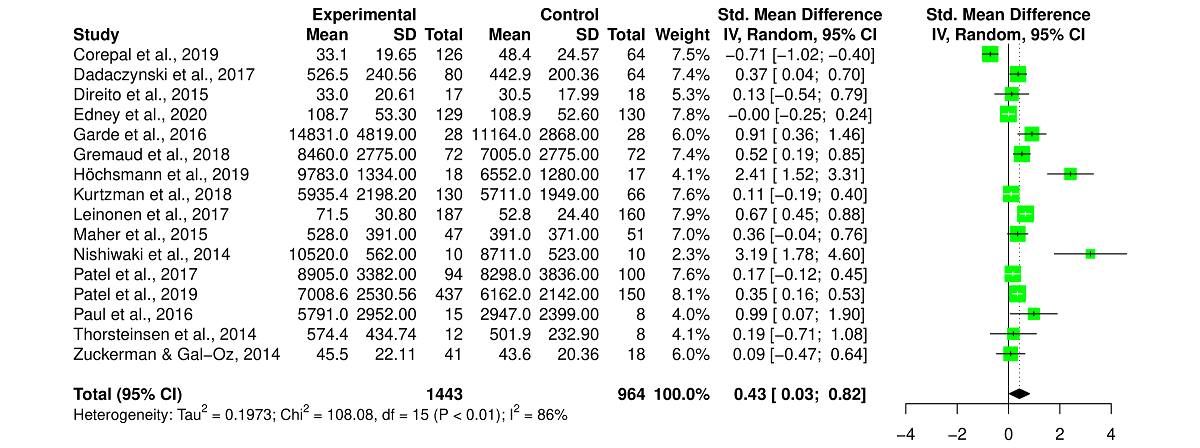
Forest plot for the effect of gamification versus control on postintervention physical activity outcomes (moderate to vigorous physical activity, daily step count, number of active minutes, and walking time). Tau-square, chi-square, and *I²* measures of between-study heterogeneity [21-36]. IV: inverse variance.

Overall, the SMD after the intervention for all PA outcomes (MVPA, daily step count, number of active minutes, and walking time) was a Hedges *g* of 0.43 (95% CI 0.03-0.82; *I^2^*=86%; Figure 2), representing a statistically significant small to medium effect. Similarly, we found a statistically significant SMD effect of a Hedges *g* of 0.38 (95% CI 0.07-0.69; *I^2^*=79%) for pre–post change scores.

Only 3 studies [23,28,31] assessed sedentary time as an outcome. Owing to this small sample size, the meta-analysis was not performed on this outcome.

### Outliers and Influential Analyses

In the first analysis, substantial statistical heterogeneity was observed. To address between-study heterogeneity, we first looked for the presence of outliers. A total of 3 studies were considered as outliers [21,25,35], and after removing them, we still obtained a significant effect of a Hedges *g* of 0.34 (95% CI 0.17-0.51) with moderate heterogeneity (*I^2^*=58%).

Leave-one-out analyses showed that sequential removal of each study did not have an important impact on the general effect size, with effect sizes ranging from a Hedges *g* of 0.33 (95% CI 0.00-0.66; *I^2^*=84%) to a Hedges *g* of 0.48 (95% CI 0.13-0.83; *I^2^*=78%; Figure S2 in Multimedia Appendix 1).

The Baujat plot (Figure S3 in Multimedia Appendix 1) shows that 4 studies explained more heterogeneity than the others, more specifically, the study by Corepal et al [21] with a heterogeneity contribution of 40.13 and an effect size influence of 3.27.

Therefore, we excluded studies with a high or unclear risk of bias. After doing so, the effect was not significant (Hedges *g*=0.33, 95% CI –0.16 to 0.81; *I^2^*=78%).

The inclusion of crossover and cluster randomized trials in the meta-analysis may lead to biases. Thus, we excluded these designs from the analysis and obtained a statistically significant effect of *g*=0.49 (95% CI 0.05-0.92; *I^2^*=67%).

Finally, we decided to exclude articles by Corepal et al [21] and Nishiwaki et al [25] in the sensitivity analyses (which have been repeated afterward for each analysis) considering their influence on the pooled result, their contribution to overall heterogeneity, and their huge risk of bias (no control of clustering, no statistical consideration of clustering, and no test of baseline differences among groups in the study by Corepal et al [21] and no washout period and very low power for the study by Nishiwaki et al [25]). After doing so, we obtained a statistically significant effect of a Hedges *g* of 0.42 (95% CI 0.14-0.69; *I^2^*=74%).

### Subgroup Analyses

We found no statistical differences in the effects between studies with participants with chronic diseases or healthy participants (Cochran *Q*=0.73; *P*=.39), between adults and adolescents (Cochran *Q*=0.26; *P*=.61), between studies with objective (devices) or self-reported PA outcomes (Cochran *Q*=0.23; *P*=.63), between active or inactive control groups (Cochran *Q*=0.01; *P*=.92), and between short- and long-term interventions (less or more than 12 weeks; Cochran *Q*=0.60; *P*=.44).

When performing the sensitivity analysis, there was a statistically significant effect of intervention on PA in adults (Hedges *g*=0.36, 95% CI 0.03-0.69; *I^2^*=71%; Figure S4 in Multimedia Appendix 1), on healthy people (Hedges *g*=0.35, 95% CI 0.15-0.55; *I^2^*=63%; Figure S5 in Multimedia Appendix 1), when the PA measure was objective (Hedges *g*=0.45, 95% CI 0.08-0.82; *I^2^*=80%; Figure S6 in Multimedia Appendix 1), when the PA measure was self-reported (Hedges *g*=0.24, 95% CI 0.08-0.39; *I^2^*=0%; Figure S6 in Multimedia Appendix 1), and for short interventions of <12 weeks (equivalent to a 3-month program; Hedges *g*=0.44, 95% CI 0.19-0.69; *I^2^*=16%; Figure S7 in Multimedia Appendix 1).

Moreover, subgroup analyses allowed us to examine the effect of gamified interventions when compared with inactive control groups and active control groups. After sensitivity analyses, we found a statistically significant effect of gamified interventions, both when compared with inactive control groups (Hedges *g*=0.58, 95% CI 0.08-1.07; *I^2^*=81%; Figure S8 in Multimedia Appendix 1) and when compared with active control groups (Hedges *g*=0.23, 95% CI 0.05-0.41; *I^2^*=37%; Figure S8 in Multimedia Appendix 1).

### Meta-Regressions

The age of participants (*β*=.01, 95% CI –0.02 to 0.04; *P*=.39), their gender (*β*=.01, 95% CI –0.01 to 0.02; *P*=.47), their BMI (*β*=.04, 95% CI –0.16 to 0.09; *P*=.53), the duration of the intervention (*β*=-.01, 95% CI –0.06 to 0.04; *P*=.74), or the number of game mechanics included in the intervention (*β*=.01, 95% CI –0.17 to 0.19; *P*=.91) were not statistically significantly associated with an increase in PA.

Lack of data precluded further meta-regressions, such as comparisons of leisure PA, or test of moderators, such as the impact of social incentives or the theoretical model used to develop the intervention.

### Publication Bias

**Figure 3:**
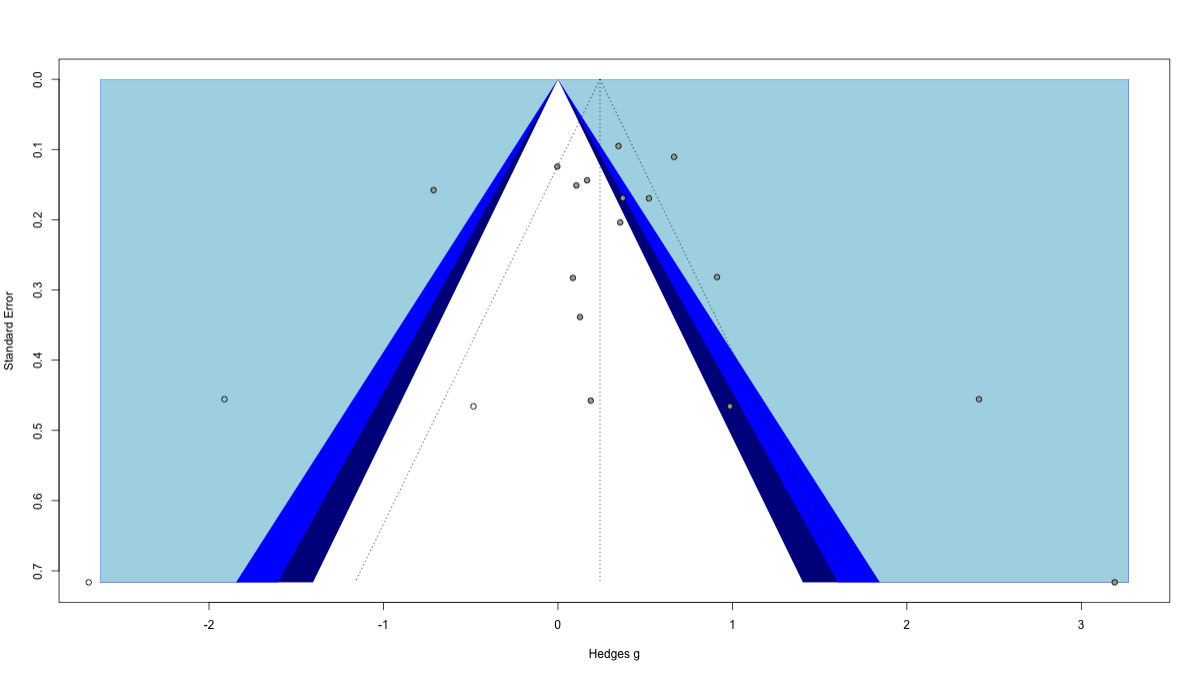
Funnel plot after trim-and-fill bias correction. A filled circle represents an included study, and an empty circle represents a missing study.

First, an inspection of the funnel plot showed that the effect sizes of individual studies were relatively symmetrically distributed around the pooled effect size. This observation was supported by the Egger test of the intercept, which indicated no asymmetry in the funnel plot (b_0_=1.38, 95% CI –0.83 to 4.77; *P*=.19). We then applied a bias-correction technique, the trim-and-fill method, which indicated that 3 studies were missing at the bottom left of the funnel plot to obtain a full symmetry. After imputing the effect sizes corresponding of these missing studies to obtain a totally symmetrical funnel plot (Figure 3), the bias corrected summary effect was of a Hedges *g* of 0.24 (95% CI –0.24 to 0.73).

**Figure 4:**
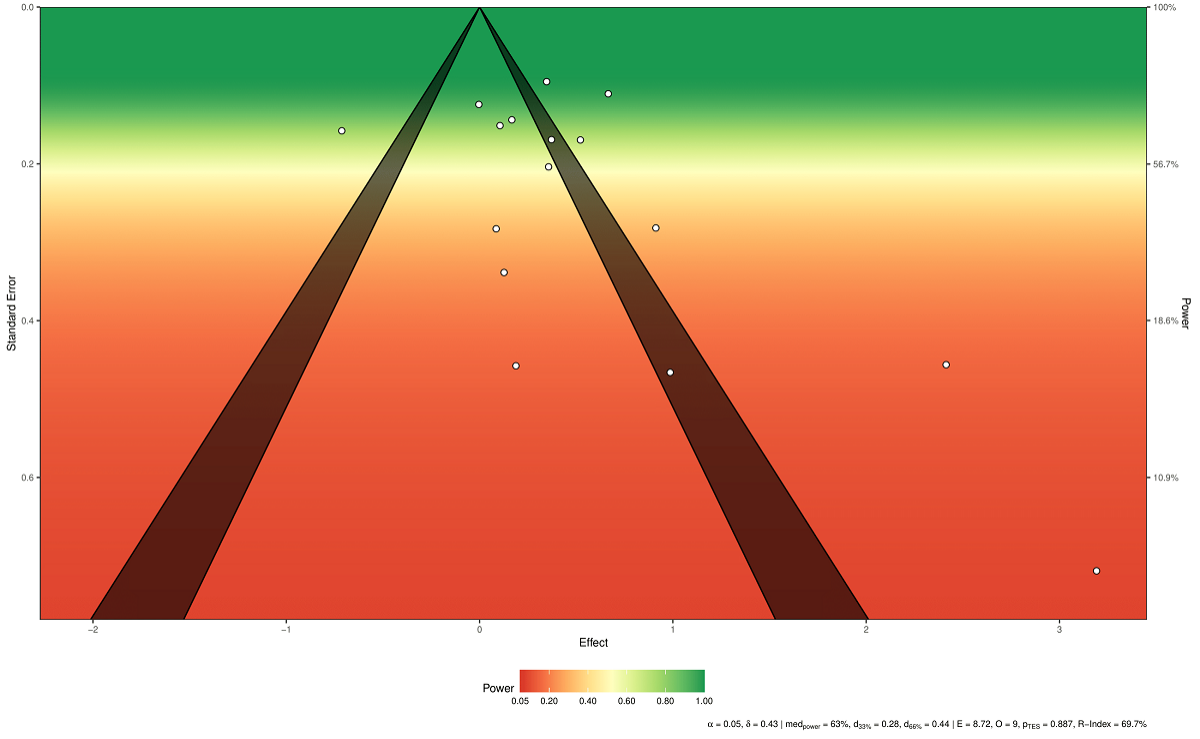
Power-enhanced funnel plot. White circles represent included studies. δ: true effect size; medpower: the median power of all tests; d33%: effect size needed for achieving 33% of median power; d66%: effect size needed for achieving 66% of median power; E: expected number of positive studies; O: observed number of positive studies; pTES: test of excess significance *P* value.

Finally, the sunset funnel plot (Figure 4) showed significant differences in power among studies, with some characterized by very low statistical power (7 studies under 45% power and 4 studies under 18%). The median power of all the tests was 63%.

### Secondary Analyses

#### Follow-up

**Figure 5:**
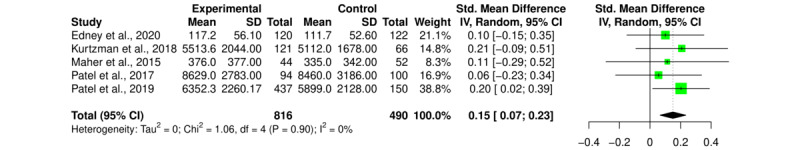
Forest plot for the effect of gamification versus control on PA outcomes (moderate to vigorous physical activity and daily step count) after a follow-up period (from 12 to 24 weeks after the end of the intervention). Tau-square, chi-square, and *I²* measures of between-study heterogeneity [24,26,27,32,36]. IV: inverse variance.

There was no statistically significant effect of gamified interventions on total PA (MVPA and daily step count) after follow-up periods with an SMD of a Hedges *g* of 0.09 (95% CI –0.07 to 0.26; *I^2^*=21%). When we performed the sensitivity analysis, gamification significantly increased PA (MVPA and daily step count) at follow-up (from 12 to 24 weeks after the end of the intervention; *g*=0.15, 95% CI 0.07-0.23; *I^2^*=0%; Figure 5).

#### Steps

**Figure 6:**
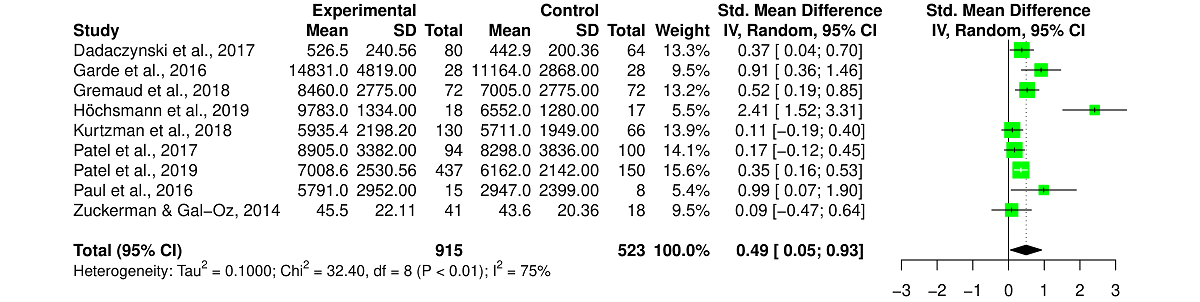
Forest plot for the effect of gamification versus control on steps outcomes (daily step count and walking time). Tau-square, chi-square, and *I²* measures of between-study heterogeneity [22,26-28,30,33-36]. IV: inverse variance.

**Figure 7:**
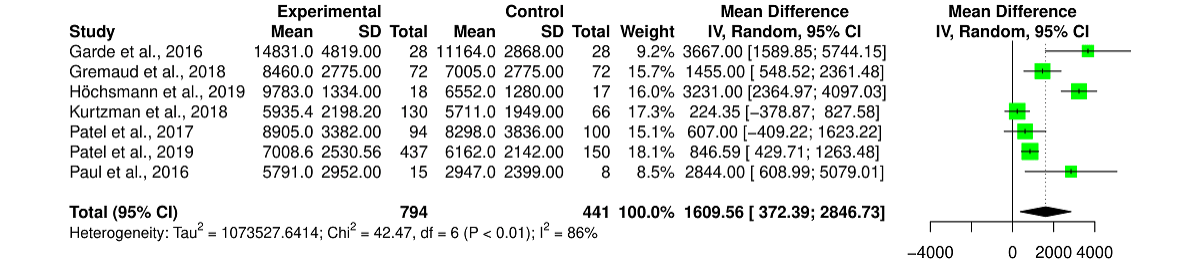
Forest plot for the mean difference of daily steps between gamification and control. Tau-square, chi-square, and *I²* measures of between-study heterogeneity [26-28,33-36]. IV: inverse variance.

We found no statistically significant effect of gamified interventions on step outcomes with an SMD of a Hedges *g* of 0.53 (95% CI –0.09 to 1.15; *I^2^*=89%), but a significant improvement in the number of daily steps with an MD of +1420.57 steps per day (95% CI 435.41-2405.73; *I^2^*=95%) was observed. When excluding the 2 studies in the sensitivity analysis, we obtained a statistically significant effect of gamification on daily steps of a Hedges *g* of 0.49 (95% CI 0.05-0.93; *I^2^*=75%; Figure 6) and a statistically significant MD of +1609.56 steps per day (95% CI 372.39-2846.73; *I^2^*=86%; Figure 7).

#### Moderate to Vigorous PA

**Figure 8:**
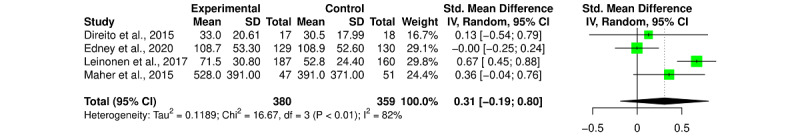
Forest plot for the effect of gamification versus control on moderate to vigorous physical activity. Tau-square, chi-square, and *I²* measures of between-study heterogeneity [23,24,31,32]. IV: inverse variance.

There was no statistically significant effect of gamification on MVPA with an SMD of a Hedges *g* of 0.09 (95% CI –0.57 to 0.74; *I^2^*=93%). There was no statistically significant effect of a Hedges *g* of 0.31 (95% CI –0.19 to 0.80; *I^2^*=82%) in the sensitivity analysis (Figure 8).

### Summary of Findings (Grading of Recommendations Assessment, Development, and Evaluation)

The quality of evidence (grading of recommendations assessment, development, and evaluation, [GRADE]) in the included studies after sensitivity analyses for short-term PA, long-term PA, MVPA, steps, and daily steps was scored from high to low (Table 2). The quality was downgraded for some outcomes because of high heterogeneity, high risk of bias, or imprecision owing to large CIs. Summaries of the various meta-analysis conducted in this review both on postintervention scores and pre–post intervention change scores are presented in Multimedia Appendix 1 (Figures S9-S11).

**Table 2:** Summary of findings.

Outcome	Number of participants (number of studies)	Standardized mean difference or mean difference (95% CI)	Quality of evidence (grading of recommendations assessment, development, and evaluation)
General PA^a^	2197 (14)	0.42 (0.14 to 0.69)	Low^b,c,d^
General PA (in comparison with active control groups)	1485 (7)	0.23 (0.05 to 0.41)	High
Long-term PA (follow-up)	1306 (5)	0.15 (0.07 to 0.23)	High
MVPA^e^	739 (4)	0.31 (–0.19 to 0.80)	Low^b,c,d^
Steps	1438 (9)	0.49 (0.05 to 0.93)	Low^b,c,d^
Daily steps	1235 (7)	1609.56 (372.39 to 2846.73)	Moderate^b,d^

^a^PA: physical activity.

^b^Downgraded because of high heterogeneity.

^c^Downgraded because of risks of bias.

^d^Downgraded because of imprecision (large CIs).

^e^MVPA: moderate to vigorous physical activity.

## Discussion

### Principal Findings

#### Summary Effect

This meta-analysis of RCTs, including 16 studies and 2407 participants, revealed a statistically significant effect of gamified interventions, on average, of 12 weeks on total PA (Hedges *g*=0.42, 95% CI 0.14-0.69 after sensitivity analyses). This effect was small to medium, suggesting the effectiveness of gamified interventions in promoting PA in both healthy participants and participants with chronic diseases. This significant effect was robust, as it persisted even after the different influence analyses were performed. Moreover, the effect was statistically significant both for objective measures of PA (Hedges *g*=0.45, 95% CI 0.08-0.82) and self-reported measures (Hedges *g*=0.24, 95% CI 0.08-0.39) after sensitivity analyses. Unsurprisingly, subgroup analyses revealed after sensitivity analyses that the effect of gamified interventions is greater when compared with inactive control groups (such as waiting lists) than when compared with active control groups benefiting from a nongamified intervention (Hedges *g*=0.58, 95% CI 0.08-1.07 vs Hedges *g*=0.23, 95% CI 0.05-0.41). Nevertheless, these effects were both statistically significant. This suggests that gamified interventions are not only efficient in changing behavior but also, to a lesser extent, effective compared with equivalent nongamified PA interventions (such as smartphone apps or self-monitoring interventions). These results are important considering the assets of gamification, which has the advantages of (1) reorganizing existing activity rather than adding additional demands to people’s lives [13], (2) being easily implemented in natural contexts, and (3) having a broad accessibility through technology and advancing sensors, permitting to address a large population.

#### Long-term Effect

When we analyzed the long-term effect of these interventions based on the follow-up measures of PA, carried out from 12 to 24 weeks (mean 14.4 weeks) after the end of the intervention, we found a statistically significant very small to small effect size of a Hedges *g* of 0.15 (95% CI 0.07-0.23) after sensitivity analyses. These results indicate that the effect of gamification persists after the end of the program, suggesting that it is not just a novelty effect due to the playful nature of gamification. However, this long-term effect was weaker and decreased with time after the end of the intervention.

#### Generalizability of Gamified Interventions

The absence of subgroup differences or effects of age, gender, and BMI on the pooled effect suggests a good generalizability of gamified interventions, which can be used for several types of populations. Thus, gamification may not only be efficient in young healthy individuals but can also target any kind of population regardless of their age or health status.

In sum, gamified interventions appear as an efficient tool to improve the PA of various populations, with moderate superiority over other similar interventions, such as mobile health monitoring apps, and a moderate sustainability of the effect after the intervention. Nevertheless, if many PA interventions increase PA levels in the short term, translating these temporary changes into long-term PA participation continues to be a challenge for PA research [59]. With that in mind, the potential of gamification for PA increases in the long term, even minimal, is particularly important and promising in the area of PA interventions.

### Additional Findings

#### Effect of Gamification on the Step Count

If the overall effect of gamified interventions on PA is positive, they increase the step count more than MVPA. Indeed, after sensitivity analyses, on the one hand, the meta-analyses revealed a statistically significant effect (Hedges *g*=0.49, 95% CI 0.05-0.93) of gamification for steps outcomes, with a statistically significant improvement of 1609.56 steps per day (95% CI 372.39-2846.73) for participants benefiting from gamified intervention versus those in the control group. On the other hand, no statistically significant effect of gamified interventions on MVPA was found (Hedges *g*=0.31, 95% CI –0.19 to 0.80). This can be explained by the game metrics and mechanics of the interventions included in the review, which are mainly focused on the step count of participants. Few interventions directly targeted MVPA. In the included studies, only 2 interventions [31,35] integrated multi-PA intensity goals and mechanics, notably with physical exercises or running sessions in the game. In other words, participants played most of the time with their number of steps and had to generally walk more to make points and play the game. This results in an increase in walking time but not necessarily in more intense PA.

These findings are interesting considering the potential health benefits of increasing the number of daily steps by 1600 because of gamification. Indeed, previous work showed that walking was statistically associated with decline in all-cause and cardiovascular mortality [60-63] and an improvement in body composition [64]. Moreover, Oja et al [62] suggested that any walking exposure is beneficial for cardiovascular health, endorsing the idea that the most important is more global PA regardless of the intensity [7,65] even when this activity only includes walking [60]. In comparison, a previous study evaluating the effectiveness of activity trackers with and without incentives to increase PA [66] showed a significant improvement of 1050 daily steps for the cash incentive intervention versus the control intervention (95% CI 600-1490) but no statistically significant difference for the Fitbit-only group (340 daily steps, 95% CI –100 to 790]). In light of these results, gamified interventions appear as an added value compared with current interventions. Considering that 40% of the volunteers in this study abandoned their Fitbit monitor within 6 months, gamification is also a way to keep participants involved and motivated within the intervention.

#### Duration of Intervention

Our meta-regression analysis did not find an association between the observed effect of gamification on PA and intervention length. However, although no statistically significant effect of gamification for an intervention length of ≥12 weeks was found (Hedges *g*=0.41, 95% CI –0.19 to 1.01), the meta-analysis revealed a statistically significant effect of gamified interventions of <12 weeks on global PA (Hedges *g*=0.44, 95% CI 0.19-0.69). According to a previous meta-analysis that reported significant positive effects of smartphone apps on PA only when used over a short-term period of <3 months [67], these results suggest that a condensed intervention could benefit more than a longer one, which could become redundant, boring, and exhausting for participants in the long run.

#### Statistical Heterogeneity

The meta-analysis also revealed considerable statistical heterogeneity. This heterogeneity may be explained by differences in study quality, diversity of designs, and variations in study populations. Despite several subgroup analyses, we cannot rule out that these subgroups and characteristics may not explain all the variance of the interventions. Indeed, demographic data often do not fully explain the differences in the effectiveness of interventions [68,69], and more precise sociopsychological variables such as personality traits or motivational factors could explain this poorly understood significant heterogeneity. Moreover, the risk of bias analysis and the sunset funnel plot showed substantial differences in the quality of the included studies that can influence the heterogeneity. Finally, various trial designs were used (ie, parallel RCT, cluster RCT, and crossover RCT) that can also contribute to the overall statistical heterogeneity.

### Better Understanding Gamification Mechanisms

This meta-analysis is informative regarding the effectiveness of gamified interventions. In view of the observed heterogeneity, the next step will be to investigate its causes from an interventional and theoretical perspective. Gamified interventions involve multiple interacting elements, and it is crucial to estimate the weight of each element in the behavior change process and how they interact with each other. Is it game mechanics, the implementation of behavior change techniques, or the presence of social interactions that make gamification effective? Unfortunately, the small number of studies included in the meta-analysis impeded us from conducting in-depth moderation analyses to answer this question. To better understand these relations, it is essential that both the development and assessment of gamified interventions be central, transparent, evidence-based, context-aware, and research-oriented [70]. Moreover, if theoretical psychological models are often mentioned in the introduction of articles included in the review (Table 1), few have investigated the psychological mechanisms of their interventions in the field [22,31,71]. Future studies should explicitly discuss motivational theory and systematically test the effect of gamification on psychological outcomes known to be involved in behavior change (eg, self-efficacy, attitudes, and intention) to better understand its mechanisms. The consideration of personality traits and psychological variables to determine behavioral phenotypes [68] is a promising way to evaluate participant’s responses to the interventions.

### Perspectives for Future Research and Implications for Practice

The findings from this meta-analysis allow us to draw and discuss future work concerning the gamification of PA and SB. First, future trials should be conducted with more adequately powered sample sizes and should be strictly multiple arms–RCTs to isolate the effects of gamification elements and better understand gamification mechanisms. Second, the long-term effects are currently the main challenge of health interventions. Thus, it is essential to investigate the evolution of the effects of gamified interventions over time. Therefore, there is a need for more long-term follow-up measurements. In addition, the potentialities of digital technologies and their capacity to collect a large amount of real-world data could be used to assess the evolution patterns of the effect, allowing the detailed identification of its sustainability and evolvement or even make forecasts via time series analyses. Third, to our knowledge, only 1 team of researchers worked on a gamified intervention targeting SB by introducing sedentary breaks as a gaming part [72]. Following this line of research, it could be interesting to develop gamified interventions affording participants to take more sedentary breaks. Finally, the cost-effectiveness ratio of gamified interventions may be better than that of many current interventions, considering the ease of implementation and generalizability of gamification. However, this assertion will have to be tested in future trials, including economic analyses.

In light of our results, gamified interventions appear to be a promising avenue to promote PA in different populations both in prevention in healthy people and in the treatment of chronic diseases. Gamified interventions have many benefits for participants with chronic diseases, such as empowerment of participants by improving their self-management skills, an effect across broad audiences enabling to target different types of pathologies, and an everyday life fit and easy implementation. Similar to other digital health processes, gamification makes it possible to address more patients, especially those who are isolated from health care facilities. Importantly, gamified interventions are especially pertinent during a health pandemic, such as the COVID-19 outbreak, in which PAs and social interactions are restricted because of lockdown or teleworking and where structured PA possibilities are limited both indoors and outdoors. Gamifying walking and daily activities is, in this context, a great way to improve PA and limit SB of individuals in addition to providing social interaction among players. In the meantime, the face-to-face management of chronic diseases is usually suspended during the pandemic, which underlines the importance of offering remote supervision of PA.

Nevertheless, in view of the weaker postintervention effect, this study suggests that a one-shot intervention is not sufficient. A more interesting design would be to address multiple *gamification doses* during or after the course of treatment to obtain a sustainable implementation of the PA behavior. This configuration would also provide an ideal duration of intervention to avoid exhausting the participants with gamified interventions for >12 weeks.

### Strengths and Limitations

To our knowledge, this is the first meta-analysis to quantitatively evaluate the effects of gamification on PA. This review has several other strengths. First, we conducted a comprehensive search strategy using multiple databases in collaboration with an academic librarian. Second, all stages of the review (screening and data extraction) were independently realized by 2 reviewers. Finally, various novel publication bias analyses and influence analyses were conducted in parallel with different subgroup analyses and meta-regressions.

However, some limitations of this work must be mentioned. Overall, the meta-analysis included a small number of studies, and some articles were feasibility or pilot trials. Therefore, several trials included small sample sizes and were highly underpowered. Some studies were conducted with a high risk of bias. One of the main limitations of this work is the impossibility of demonstrating that the effect of gamified interventions is led by gamification itself given the lack of research examining this question. Finally, in the main analysis, we included diverse PA outcomes evaluating similar constructs but which are slightly different in practice. Moreover, not all included outcomes were objectively measured. As the field matures and new trials are published, an update of this work will be important to confirm these preliminary results.

### Conclusions

To conclude, gamified interventions appear to be a promising avenue for promoting PA in various populations. Influencing primarily the number of daily steps of the participants, gamification is an interesting way to improve daily PA and appears more efficient than equivalent nongamified interventions, such as mobile health apps. However, if the effect of gamification persists during follow-up, suggesting that gamified interventions are more than a novelty effect, this effect decreases with time with a smaller long-term effect. The integration of gamification in more global health care interventions could be a way to address this limited sustainability. Future rigorous trials are required to explore these perspectives.

## Appendix

Multimedia Appendix 1 Supplementary materials.

Multimedia Appendix 2 Risk of bias summary for studies included in the meta-analysis.

Multimedia Appendix 3 Risk of bias graph: review authors' judgments about each risk of bias item presented as percentages across all included studies.

## Abbreviations

GRADE: grading of recommendations assessment, development, and evaluation
MD: mean difference
MET: metabolic equivalent of task
MVPA: moderate to vigorous physical activity
OSF: Open Science Framework
PA: physical activity
PRISMA: Preferred Reporting Items for Systematic Reviews and Meta-Analyses
RCT: randomized controlled trial
SB: sedentary behavior
SMD: standardized mean difference
