# Supplementary material for: Evaluating the Effectiveness of Gamification on Physical Activity: Systematic Review and Meta-analysis of Randomized Controlled Trials
Source: J Med Internet Res. 2022 Jan 4;24(1):e26779. doi: 10.2196/26779 (PMC8767479; doi:10.2196/26779)
Supplement: Multimedia Appendix 1 [file jmir_v24i1e26779_app1.pdf]

Journal of Medical Internet Research

# Evaluating the Effectiveness of Gamification on Physical Activity: Systematic Review and Meta-analysis of Randomized Controlled Trials

Alexandre Mazéas*, Martine Duclos, Bruno Pereira, Aïna Chalabaev

## Multimedia Appendix 1:

## Supplementary Materials

1. Supplemental figures referred in the manuscript
2. Search equations for databases

**All Data and materials are available on the OSF page of the project** (https://osf.io/x2483/)

*Corresponding author: UFR STAPS, 1741 rue de la Piscine, 38400 Saint Martin d'Hères, France. Email: alexandre.mazeas@univ-grenoble-alpes.fr ; @AlexMazeas on Twitter.

# 1. Supplemental figures referred in the manuscript

**Table S1** Keywords used for database searches

| Physical activity and sedentary behaviors | Gamification | e-health | | Trial |
|---|---|---|---|---|
| exercise | | telemedicine | iOS | |
| sport | gamification | telehealth | android | “randomized |
| “active living” | exergaming | e-health | website | controlled |
| “active transport” | game | m-health | digital* | trial” |
| “exercise therapy” | gamified | mobile | electronic* | RCT |
| fitness | gaming | internet | device | randomized |
| sedent* | gameful* | applications | framework* | trial |
| “sitting time” | multiplayer | smartphone | wearable | “clinical trial” |
| inactive | player | cellphone | “social | “controlled |
| inactivity | playing | Iphone | network” | trial” |
| walk* | | facebook | Technolog* | |

**N.B.** Some additional terms and synonyms could be used for some database. For more information, see search strings for each database.

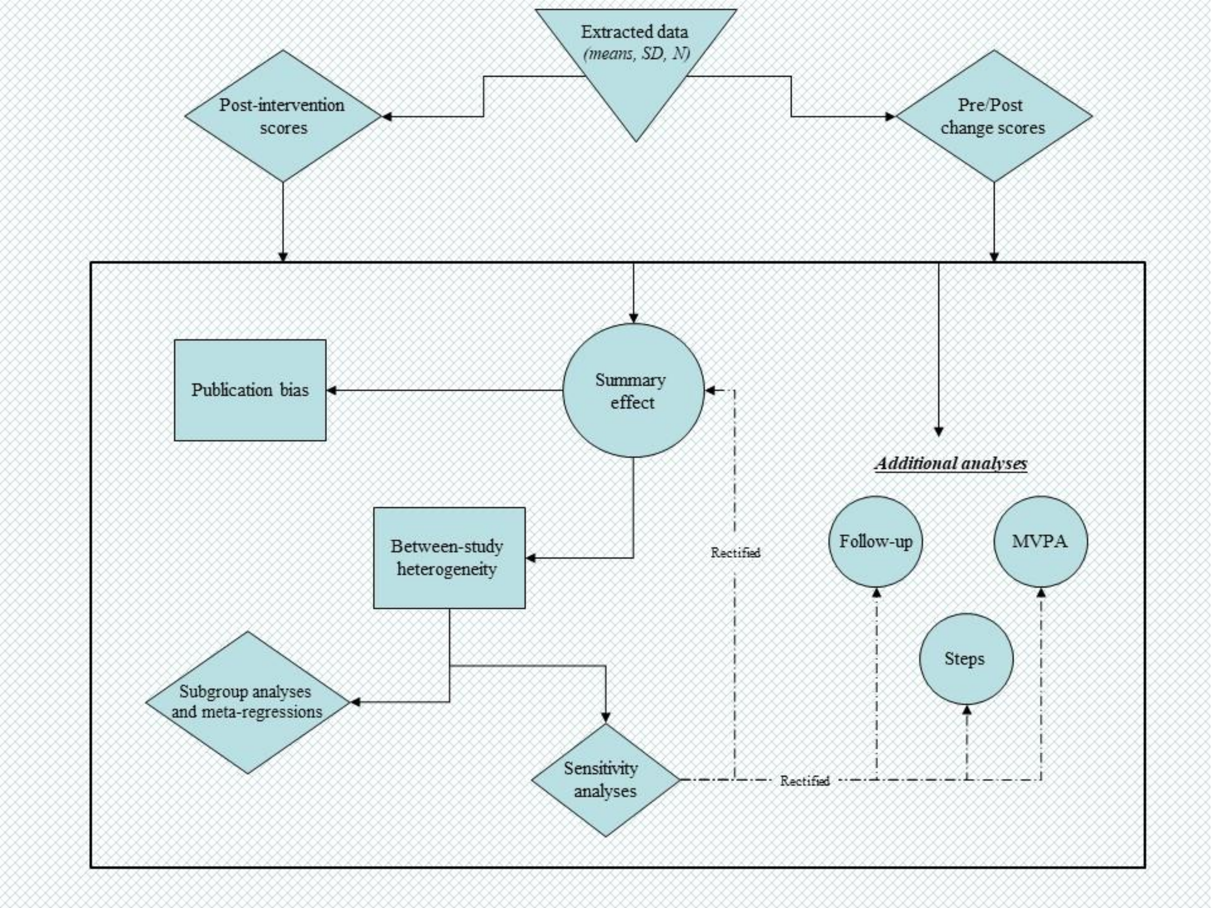


**Fig. S1** Statistical analyses summary. All analyses were conducted two times, both for post-intervention scores and for pre/post intervention change scores

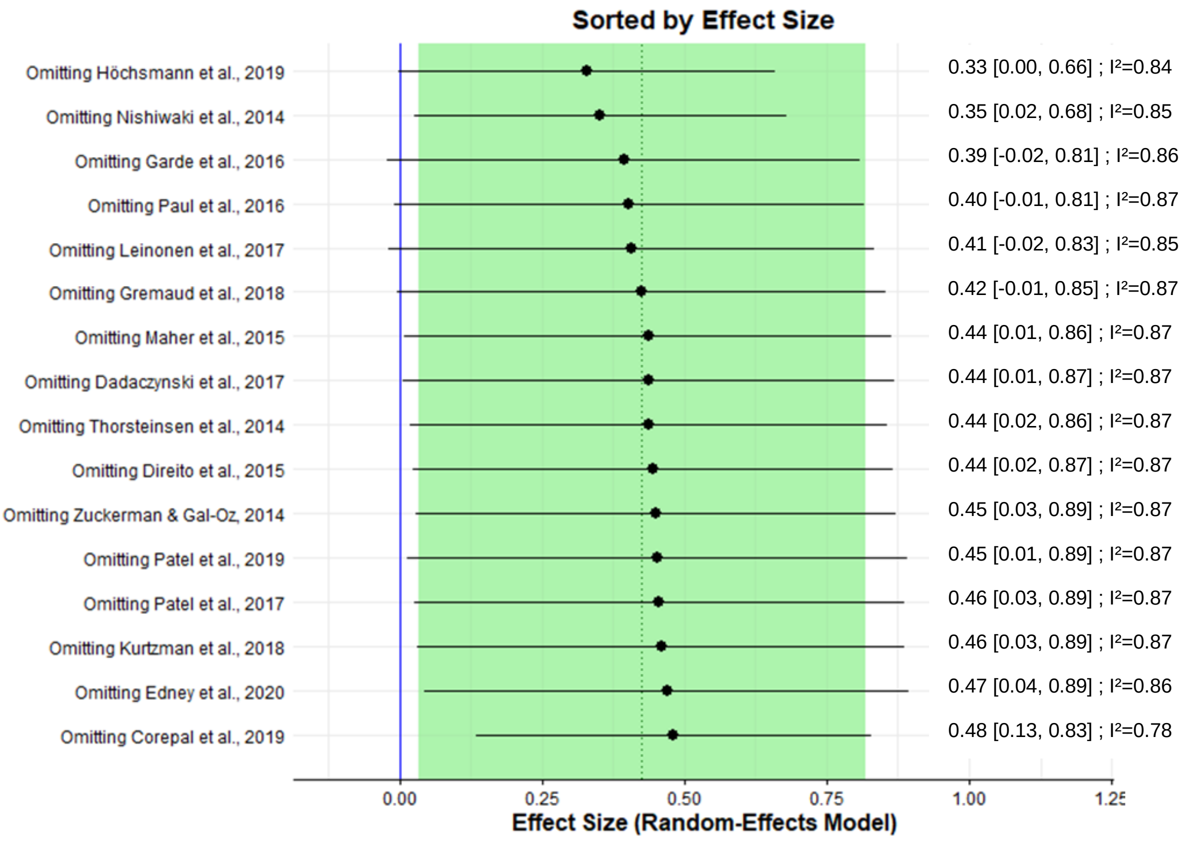


**Fig. S2** Forest plots representing Leave-one-out analyses ordered by effect size (Hedge's *g*; low to high)

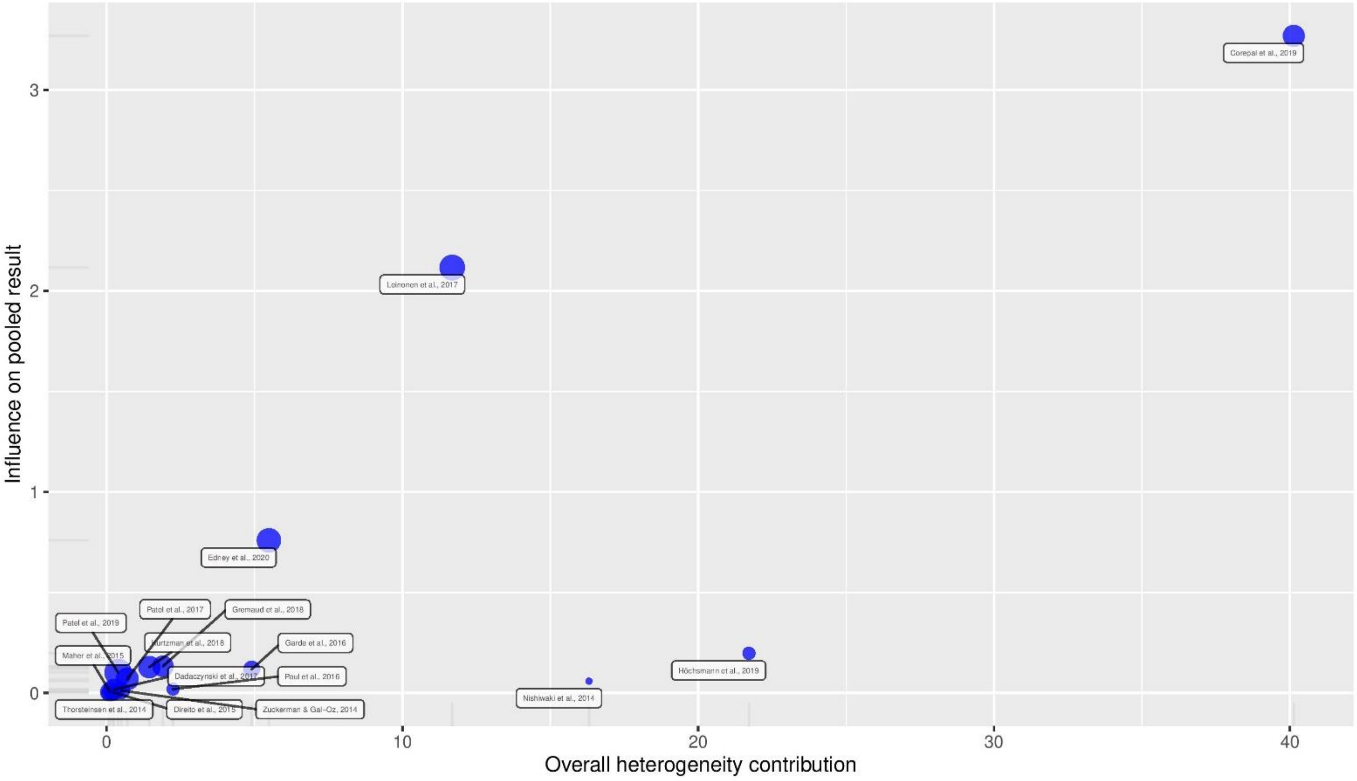


**Fig. S3** Baujat plot representing the contribution of each study to the overall heterogeneity (measured by Cochran's Q) in function of its influence on the pooled effect size

| Study or Subgroup | Experimental Mean | SD | Total | Control Mean | SD | Total | Weight | Std. Mean Difference IV, Random, 95% CI |
|---|---|---|---|---|---|---|---|---|
| Population = Adults | | | | | | | | |
| Dadaczynski et al., 2017 | 526.5 | 240.56 | 80 | 442.9 | 200.36 | 64 | 8.6% | 0.37 [ 0.04; 0.70] |
| Edney et al., 2020 | 108.7 | 53.30 | 129 | 108.9 | 52.60 | 130 | 9.7% | -0.00 [-0.25; 0.24] |
| Gremaud et al., 2018 | 8460.0 | 2775.00 | 72 | 7005.0 | 2775.00 | 72 | 8.6% | 0.52 [ 0.19; 0.85] |
| Höchsmann et al., 2019 | 9783.0 | 1334.00 | 18 | 6552.0 | 1280.00 | 17 | 3.3% | 2.41 [ 1.52; 3.31] |
| Kurtzman et al., 2018 | 5935.4 | 2198.20 | 130 | 5711.0 | 1949.00 | 66 | 9.1% | 0.11 [-0.19; 0.40] |
| Maher et al., 2015 | 528.0 | 391.00 | 47 | 391.0 | 371.00 | 51 | 7.7% | 0.36 [-0.04; 0.76] |
| Patel et al., 2017 | 8905.0 | 3382.00 | 94 | 8298.0 | 3836.00 | 100 | 9.2% | 0.17 [-0.12; 0.45] |
| Patel et al., 2019 | 7008.6 | 2530.56 | 437 | 6162.0 | 2142.00 | 150 | 10.4% | 0.35 [ 0.16; 0.53] |
| Paul et al., 2016 | 5791.0 | 2952.00 | 15 | 2947.0 | 2399.00 | 8 | 3.2% | 0.99 [ 0.07; 1.90] |
| Thorsteinsen et al., 2014 | 574.4 | 434.74 | 12 | 501.9 | 232.90 | 8 | 3.3% | 0.19 [-0.71; 1.08] |
| Zuckerman & Gal-Oz, 2014 | 45.5 | 22.11 | 41 | 43.6 | 20.36 | 18 | 5.9% | 0.09 [-0.47; 0.64] |
| Total (95% CI) | | | 1075 | | | 684 | 79.1% | 0.36 [ 0.03; 0.69] |
| Heterogeneity: $Tau^2 = 0.0761$; $Chi^2 = 34.8$, df = 10 (P < 0.01); $I^2 = 71\%$ | | | | | | | | |
| Population = Adolescents | | | | | | | | |
| Direito et al., 2015 | 33.0 | 20.61 | 17 | 30.5 | 17.99 | 18 | 4.9% | 0.13 [-0.54; 0.79] |
| Garde et al., 2016 | 14.8 | 4.82 | 28 | 11.2 | 2.87 | 28 | 5.9% | 0.91 [ 0.36; 1.46] |
| Leinonen et al., 2017 | 71.5 | 30.80 | 187 | 52.8 | 24.40 | 160 | 10.1% | 0.67 [ 0.45; 0.88] |
| Total (95% CI) | | | 232 | | | 206 | 20.9% | 0.63 [-0.16; 1.41] |
| Heterogeneity: $Tau^2 = 0.0354$; $Chi^2 = 3.27$, df = 2 (P = 0.19); $I^2 = 39\%$ | | | | | | | | |
| **Total (95% CI)** | | | **1307** | | | **890** | **100.0%** | **0.42 [ 0.14; 0.69]** |
| Heterogeneity: $Tau^2 = 0.0846$; $Chi^2 = 49.21$, df = 13 (P < 0.01); $I^2 = 74\%$ | | | | | | | | |
| Residual heterogeneity: $Tau^2$ = NA; $Chi^2 = 38.07$, df = 12 (P < 0.01); $I^2 = 68\%$ | | | | | | | | |

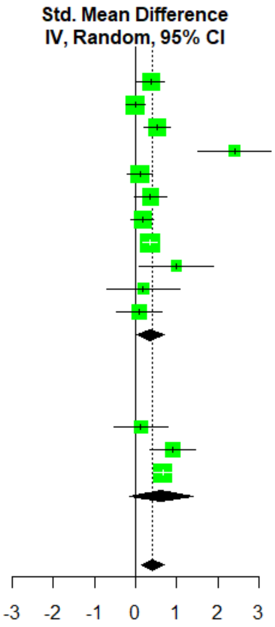


**Fig. S4** Forest plot with age subgroups (adults versus adolescents) for the effect of gamification versus control on post-intervention PA outcomes (moderate-to-vigorous physical activity

(MVPA), daily step count, number of active minutes, and walking time). *SD* standard deviation, *Std* standardized, *IV* inverse variance, *CI* confidence interval, *Tau*², *Chi*², and *I*² measures of between-study heterogeneity

| Study or Subgroup | Experimental Mean | SD | Total | Control Mean | SD | Total | Weight | Std. Mean Difference IV, Random, 95% CI |
|---|---|---|---|---|---|---|---|---|
| Pathology = No | | | | | | | | |
| Dadaczynski et al., 2017 | 526.5 | 240.56 | 80 | 442.9 | 200.36 | 64 | 8.6% | 0.37 [ 0.04; 0.70] |
| Direito et al., 2015 | 33.0 | 20.61 | 17 | 30.5 | 17.99 | 18 | 4.9% | 0.13 [-0.54; 0.79] |
| Edney et al., 2020 | 108.7 | 53.30 | 129 | 108.9 | 52.60 | 130 | 9.7% | -0.00 [-0.25; 0.24] |
| Garde et al., 2016 | 14.8 | 4.82 | 28 | 11.2 | 2.87 | 28 | 5.9% | 0.91 [ 0.36; 1.46] |
| Gremaud et al., 2018 | 8460.0 | 2775.00 | 72 | 7005.0 | 2775.00 | 72 | 8.6% | 0.52 [ 0.19; 0.85] |
| Leinonen et al., 2017 | 71.5 | 30.80 | 187 | 52.8 | 24.40 | 160 | 10.1% | 0.67 [ 0.45; 0.88] |
| Maher et al., 2015 | 528.0 | 391.00 | 47 | 391.0 | 371.00 | 51 | 7.7% | 0.36 [-0.04; 0.76] |
| Patel et al., 2017 | 8905.0 | 3382.00 | 94 | 8298.0 | 3836.00 | 100 | 9.2% | 0.17 [-0.12; 0.45] |
| Thorsteinsen et al., 2014 | 574.4 | 434.74 | 12 | 501.9 | 232.90 | 8 | 3.3% | 0.19 [-0.71; 1.08] |
| Zuckerman & Gal-Oz, 2014 | 45.5 | 22.11 | 41 | 43.6 | 20.36 | 18 | 5.9% | 0.09 [-0.47; 0.64] |
| Total (95% CI) | | | 707 | | | 649 | 74.0% | 0.35 [ 0.15; 0.55] |
| Heterogeneity: $Tau^2 = 0.0554$; $Chi^2 = 24.3$, df = 9 ($P < 0.01$); $I^2 = 63\%$ | | | | | | | | |
| Pathology = Yes | | | | | | | | |
| Höchsmann et al., 2019 | 9783.0 | 1334.00 | 18 | 6552.0 | 1280.00 | 17 | 3.3% | 2.41 [ 1.52; 3.31] |
| Kurtzman et al., 2018 | 5935.4 | 2198.20 | 130 | 5711.0 | 1949.00 | 66 | 9.1% | 0.11 [-0.19; 0.40] |
| Patel et al., 2019 | 7008.6 | 2530.56 | 437 | 6162.0 | 2142.00 | 150 | 10.4% | 0.35 [ 0.16; 0.53] |
| Paul et al., 2016 | 5791.0 | 2952.00 | 15 | 2947.0 | 2399.00 | 8 | 3.2% | 0.99 [ 0.07; 1.90] |
| Total (95% CI) | | | 600 | | | 241 | 26.0% | 0.79 [-0.77; 2.34] |
| Heterogeneity: $Tau^2 = 0.2844$; $Chi^2 = 24.91$, df = 3 ($P < 0.01$); $I^2 = 88\%$ | | | | | | | | |
| **Total (95% CI)** | | | **1307** | | | **890** | **100.0%** | **0.42 [ 0.14; 0.69]** |
| Heterogeneity: $Tau^2 = 0.0846$; $Chi^2 = 49.21$, df = 13 ($P < 0.01$); $I^2 = 74\%$ | | | | | | | | |
| Residual heterogeneity: $Tau^2$ = NA; $Chi^2 = 49.21$, df = 12 ($P < 0.01$); $I^2 = 76\%$ | | | | | | | | |

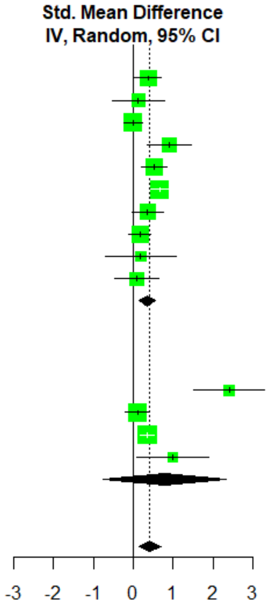


**Fig. S5** Forest plot with health condition subgroups (healthy people versus patients with chronic diseases) for the effect of gamification versus control on post-intervention PA outcomes (moderate-to-vigorous physical activity (MVPA), daily step count, number of active minutes, and walking time). *SD* standard deviation, *Std* standardized, *IV* inverse variance, *CI* confidence interval, *Tau*², *Chi*², and *I*² measures of between-study heterogeneity

| Study or Subgroup | Experimental Mean | SD | Total | Control Mean | SD | Total | Weight | Std. Mean Difference IV, Random, 95% CI |
|---|---|---|---|---|---|---|---|---|
| Measure = Self-reported | | | | | | | | |
| Dadaczynski et al., 2017 | 526.5 | 240.56 | 80 | 442.9 | 200.36 | 64 | 7.5% | 0.37 [ 0.04; 0.70] |
| Direito et al., 2016 | 2.3 | 0.53 | 17 | 2.2 | 0.62 | 18 | 4.1% | 0.10 [-0.56; 0.76] |
| Edney et al., 2020 | 432.9 | 344.40 | 129 | 384.1 | 347.30 | 130 | 8.6% | 0.14 [-0.10; 0.38] |
| Maher et al., 2015 | 528.0 | 391.00 | 47 | 391.0 | 371.00 | 51 | 6.7% | 0.36 [-0.04; 0.76] |
| Thorsteinsen et al., 2014 | 574.4 | 434.74 | 12 | 501.9 | 232.90 | 8 | 2.7% | 0.19 [-0.71; 1.08] |
| Total (95% CI) | | | 285 | | | 271 | 29.7% | 0.24 [ 0.08; 0.39] |
| Heterogeneity: $Tau^2$ = 0; $Chi^2$ = 1.75, df = 4 (P = 0.78); $I^2$ = 0% | | | | | | | | |
| Measure = Objective | | | | | | | | |
| Direito et al., 2015 | 33.0 | 20.61 | 17 | 30.5 | 17.99 | 18 | 4.1% | 0.13 [-0.54; 0.79] |
| Edney et al., 2020 | 108.7 | 53.30 | 129 | 108.9 | 52.60 | 130 | 8.7% | -0.00 [-0.25; 0.24] |
| Garde et al., 2016 | 14.8 | 4.82 | 28 | 11.2 | 2.87 | 28 | 5.1% | 0.91 [ 0.36; 1.46] |
| Gremaud et al., 2018 | 8460.0 | 2775.00 | 72 | 7005.0 | 2775.00 | 72 | 7.5% | 0.52 [ 0.19; 0.85] |
| Höchsmann et al., 2019 | 9783.0 | 1334.00 | 18 | 6552.0 | 1280.00 | 17 | 2.8% | 2.41 [ 1.52; 3.31] |
| Kurtzman et al., 2018 | 5935.4 | 2198.20 | 130 | 5711.0 | 1949.00 | 66 | 8.0% | 0.11 [-0.19; 0.40] |
| Leinonen et al., 2017 | 71.5 | 30.80 | 187 | 52.8 | 24.40 | 160 | 9.0% | 0.67 [ 0.45; 0.88] |
| Patel et al., 2017 | 8905.0 | 3382.00 | 94 | 8298.0 | 3836.00 | 100 | 8.2% | 0.17 [-0.12; 0.45] |
| Patel et al., 2019 | 7008.6 | 2530.56 | 437 | 6162.0 | 2142.00 | 150 | 9.3% | 0.35 [ 0.16; 0.53] |
| Paul et al., 2016 | 5791.0 | 2952.00 | 15 | 2947.0 | 2399.00 | 8 | 2.7% | 0.99 [ 0.07; 1.90] |
| Zuckerman & Gal-Oz, 2014 | 45.5 | 22.11 | 41 | 43.6 | 20.36 | 18 | 5.0% | 0.09 [-0.47; 0.64] |
| Total (95% CI) | | | 1168 | | | 767 | 70.3% | 0.45 [ 0.08; 0.82] |
| Heterogeneity: $Tau^2$ = 0.1088; $Chi^2$ = 49.07, df = 10 (P < 0.01); $I^2$ = 80% | | | | | | | | |
| **Total (95% CI)** | | | **1453** | | | **1038** | **100.0%** | **0.37 [ 0.14; 0.61]** |
| Heterogeneity: $Tau^2$ = 0.0744; $Chi^2$ = 52.36, df = 15 (P < 0.01); $I^2$ = 71% | | | | | | | | |
| Residual heterogeneity: $Tau^2$ = NA; $Chi^2$ = 50.82, df = 14 (P < 0.01); $I^2$ = 72% | | | | | | | | |

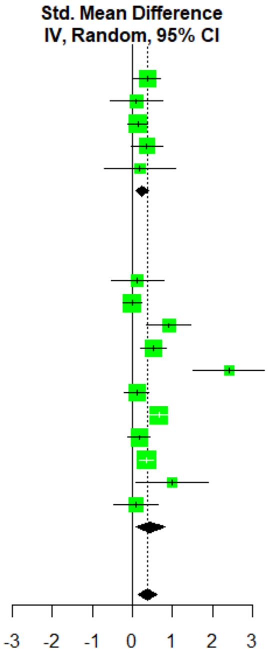


**Fig. S6** Forest plot with type of measure subgroups (self-reported versus objective measures) for the effect of gamification versus control on post-intervention PA outcomes (moderate-to-vigorous physical activity (MVPA), daily step count, number of active minutes, and walking time). *SD* standard deviation, *Std* standardized, *IV* inverse variance, *CI* confidence interval, *Tau*², *Chi*², and *I*² measures of between-study heterogeneity

| Study or Subgroup | Experimental Mean | SD | Total | Control Mean | SD | Total | Weight | Std. Mean Difference IV, Random, 95% CI |
|---|---|---|---|---|---|---|---|---|
| intervention.duration.rectif = FALSE | | | | | | | | |
| Dadaczynski et al., 2017 | 526.5 | 240.56 | 80 | 442.9 | 200.36 | 64 | 8.6% | 0.37 [ 0.04; 0.70] |
| Direito et al., 2015 | 33.0 | 20.61 | 17 | 30.5 | 17.99 | 18 | 4.9% | 0.13 [-0.54; 0.79] |
| Garde et al., 2016 | 14.8 | 4.82 | 28 | 11.2 | 2.87 | 28 | 5.9% | 0.91 [ 0.36; 1.46] |
| Gremaud et al., 2018 | 8460.0 | 2775.00 | 72 | 7005.0 | 2775.00 | 72 | 8.6% | 0.52 [ 0.19; 0.85] |
| Maher et al., 2015 | 528.0 | 391.00 | 47 | 391.0 | 371.00 | 51 | 7.7% | 0.36 [-0.04; 0.76] |
| Paul et al., 2016 | 5791.0 | 2952.00 | 15 | 2947.0 | 2399.00 | 8 | 3.2% | 0.99 [ 0.07; 1.90] |
| Zuckerman & Gal-Oz, 2014 | 45.5 | 22.11 | 41 | 43.6 | 20.36 | 18 | 5.9% | 0.09 [-0.47; 0.64] |
| Total (95% CI) | | | 300 | | | 259 | 44.9% | 0.44 [ 0.19; 0.69] |
| Heterogeneity: $Tau^2$ = 0.0109; $Chi^2$ = 7.16, df = 6 (P = 0.31); $I^2$ = 16% | | | | | | | | |
| intervention.duration.rectif = TRUE | | | | | | | | |
| Edney et al., 2020 | 108.7 | 53.30 | 129 | 108.9 | 52.60 | 130 | 9.7% | -0.00 [-0.25; 0.24] |
| Höchsmann et al., 2019 | 9783.0 | 1334.00 | 18 | 6552.0 | 1280.00 | 17 | 3.3% | 2.41 [ 1.52; 3.31] |
| Kurtzman et al., 2018 | 5935.4 | 2198.20 | 130 | 5711.0 | 1949.00 | 66 | 9.1% | 0.11 [-0.19; 0.40] |
| Leinonen et al., 2017 | 71.5 | 30.80 | 187 | 52.8 | 24.40 | 160 | 10.1% | 0.67 [ 0.45; 0.88] |
| Patel et al., 2017 | 8905.0 | 3382.00 | 94 | 8298.0 | 3836.00 | 100 | 9.2% | 0.17 [-0.12; 0.45] |
| Patel et al., 2019 | 7008.6 | 2530.56 | 437 | 6162.0 | 2142.00 | 150 | 10.4% | 0.35 [ 0.16; 0.53] |
| Thorsteinsen et al., 2014 | 574.4 | 434.74 | 12 | 501.9 | 232.90 | 8 | 3.3% | 0.19 [-0.71; 1.08] |
| Total (95% CI) | | | 1007 | | | 631 | 55.1% | 0.41 [-0.19; 1.01] |
| Heterogeneity: $Tau^2$ = 0.1236; $Chi^2$ = 40.88, df = 6 (P < 0.01); $I^2$ = 85% | | | | | | | | |
| **Total (95% CI)** | | | **1307** | | | **890** | **100.0%** | **0.42 [ 0.14; 0.69]** |
| Heterogeneity: $Tau^2$ = 0.0846; $Chi^2$ = 49.21, df = 13 (P < 0.01); $I^2$ = 74% | | | | | | | | |
| Residual heterogeneity: $Tau^2$ = NA; $Chi^2$ = 48.04, df = 12 (P < 0.01); $I^2$ = 75% | | | | | | | | |

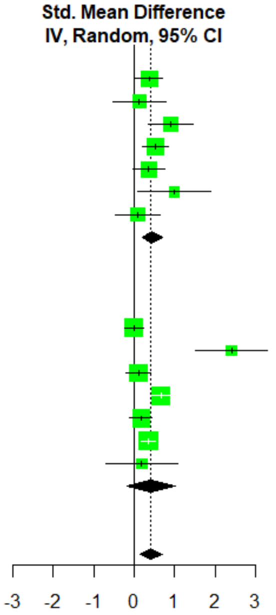


**Fig. S7** Forest plot with subgroups in function of intervention duration (less than 12 weeks versus 12 weeks or more) for the effect of gamification versus control on post-intervention PA outcomes (moderate-to-vigorous physical activity (MVPA), daily step count, number of active

minutes, and walking time). *SD* standard deviation, *Std* standardized, *IV* inverse variance, *CI* confidence interval, *Tau*², *Chi*², and *I*² measures of between-study heterogeneity

| Study or Subgroup | Experimental Mean | SD | Total | Control Mean | SD | Total | Weight | Std. Mean Difference IV, Random, 95% CI |
|---|---|---|---|---|---|---|---|---|
| **Control = Inactive** | | | | | | | | |
| Dadaczynski et al., 2017 | 526.5 | 240.56 | 80 | 442.9 | 200.36 | 64 | 7.5% | 0.37 [ 0.04; 0.70] |
| Direito et al., 2015 | 33.0 | 20.61 | 17 | 30.5 | 17.99 | 18 | 4.2% | 0.13 [-0.54; 0.79] |
| Edney et al., 2020 | 108.7 | 53.30 | 129 | 108.9 | 52.60 | 130 | 8.5% | -0.00 [-0.25; 0.24] |
| Garde et al., 2016 | 14.8 | 4.82 | 28 | 11.2 | 2.87 | 28 | 5.2% | 0.91 [ 0.36; 1.46] |
| Höchsmann et al., 2019 | 9783.0 | 1334.00 | 18 | 6552.0 | 1280.00 | 17 | 2.9% | 2.41 [ 1.52; 3.31] |
| Leinonen et al., 2017 | 71.5 | 30.80 | 187 | 52.8 | 24.40 | 160 | 8.8% | 0.67 [ 0.45; 0.88] |
| Maher et al., 2015 | 528.0 | 391.00 | 47 | 391.0 | 371.00 | 51 | 6.7% | 0.36 [-0.04; 0.76] |
| Paul et al., 2016 | 5791.0 | 2952.00 | 15 | 2947.0 | 2399.00 | 8 | 2.8% | 0.99 [ 0.07; 1.90] |
| Thorsteinsen et al., 2014 | 574.4 | 434.74 | 12 | 501.9 | 232.90 | 8 | 2.9% | 0.19 [-0.71; 1.08] |
| Total (95% CI) | | | 533 | | | 484 | 49.6% | 0.58 [ 0.08; 1.07] |
| Heterogeneity: $Tau^2$ = 0.1779; $Chi^2$ = 41.28, df = 8 (P < 0.01); $I^2$ = 81% | | | | | | | | |
| **Control = Active** | | | | | | | | |
| Direito et al., 2015 | 33.0 | 20.61 | 17 | 23.3 | 14.04 | 16 | 4.0% | 0.53 [-0.16; 1.23] |
| Edney et al., 2020 | 108.7 | 53.30 | 129 | 107.6 | 88.90 | 143 | 8.6% | 0.01 [-0.22; 0.25] |
| Gremaud et al., 2018 | 8460.0 | 2775.00 | 72 | 7005.0 | 2775.00 | 72 | 7.5% | 0.52 [ 0.19; 0.85] |
| Kurtzman et al., 2018 | 5935.4 | 2198.20 | 130 | 5711.0 | 1949.00 | 66 | 7.9% | 0.11 [-0.19; 0.40] |
| Patel et al., 2017 | 8905.0 | 3382.00 | 94 | 8298.0 | 3836.00 | 100 | 8.1% | 0.17 [-0.12; 0.45] |
| Patel et al., 2019 | 7008.6 | 2530.56 | 437 | 6162.0 | 2142.00 | 150 | 9.1% | 0.35 [ 0.16; 0.53] |
| Zuckerman & Gal-Oz, 2014 | 45.5 | 22.11 | 41 | 43.6 | 20.36 | 18 | 5.1% | 0.09 [-0.47; 0.64] |
| Total (95% CI) | | | 920 | | | 565 | 50.4% | 0.23 [ 0.05; 0.41] |
| Heterogeneity: $Tau^2$ = 0.0135; $Chi^2$ = 9.46, df = 6 (P = 0.15); $I^2$ = 37% | | | | | | | | |
| **Total (95% CI)** | | | **1453** | | | **1049** | **100.0%** | **0.39 [ 0.14; 0.63]** |
| Heterogeneity: $Tau^2$ = 0.0826; $Chi^2$ = 56.59, df = 15 (P < 0.01); $I^2$ = 73% | | | | | | | | |
| Residual heterogeneity: $Tau^2$ = NA; $Chi^2$ = 50.75, df = 14 (P < 0.01); $I^2$ = 72% | | | | | | | | |

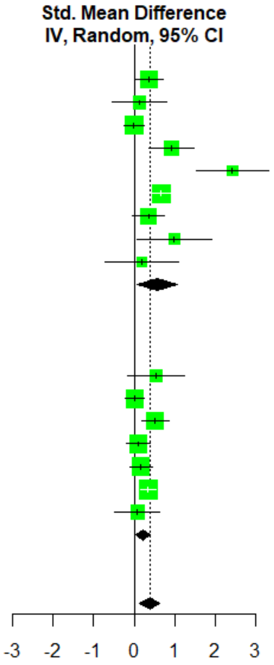


**Fig. S8** Forest plot with control groups subgroups (inactive control group versus active control group) for the effect of gamification versus control on post-intervention PA outcomes (moderate-to-vigorous physical activity (MVPA), daily step count, number of active minutes, and walking time). *SD* standard deviation, *Std* standardized, *IV* inverse variance, *CI* confidence interval, *Tau*², *Chi*², and *I*² measures of between-study heterogeneity

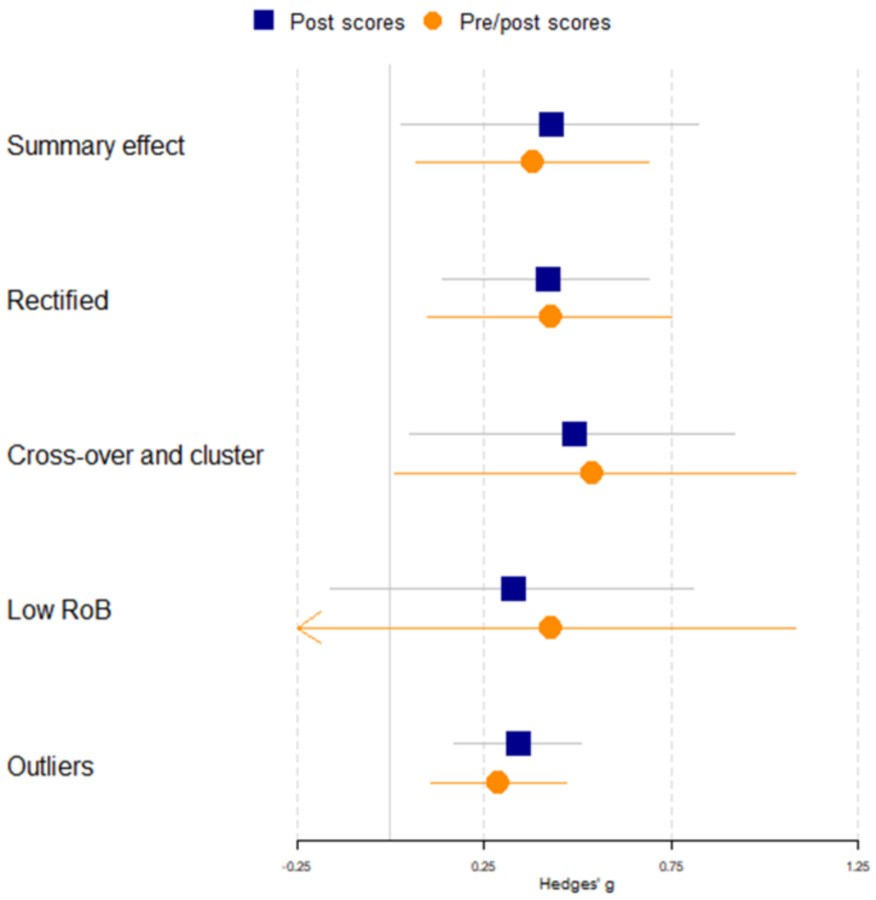


**Fig. S9** Summary of the meta-analyses conducted on global PA (i.e., summary effect of gamification versus control, rectified effect after 'sensitivity analysis', effect after removing cross-over and cluster trials, effect after removing studies with a high Risk of Bias (RoB), and effect after removing outliers) both for post-intervention scores and pre/post intervention change scores

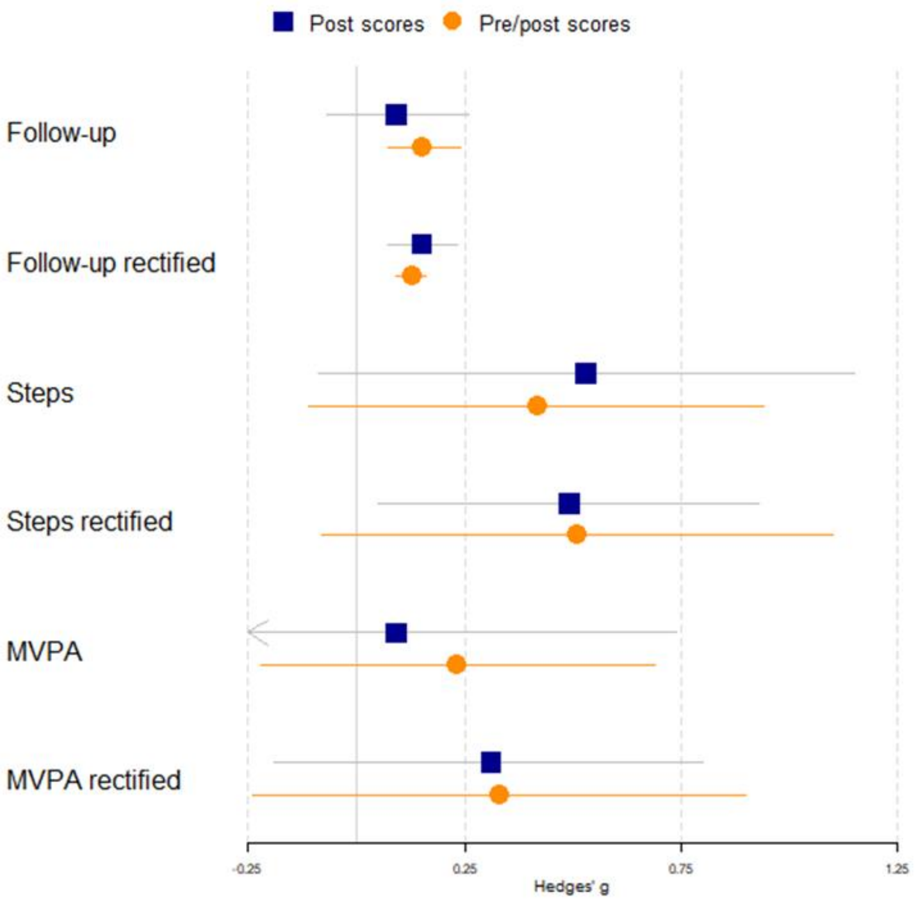


**Fig. S10** Summary of the meta-analyses conducted on other outcomes (i.e., effect of gamification versus control after a follow-up period (from 12 to 24 weeks after the end of the intervention), effect on steps outcomes, effect on moderate-to-vigorous physical activity (MVPA), and rectified effects after 'sensitivity analyses') both for post-intervention scores and pre/post intervention change scores

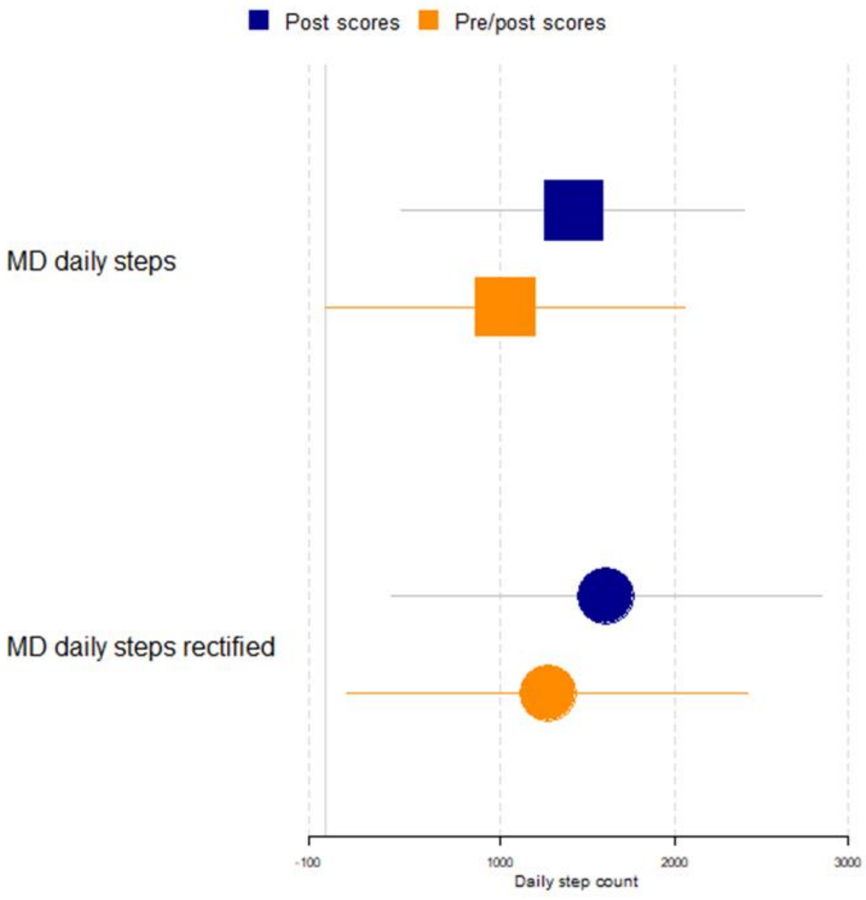


**Fig. S11** Summary of the meta-analyses conducted on daily steps (i.e., mean difference of daily steps between gamification and control) both for post-intervention scores and pre/post intervention change scores

## 2. Search equations for databases

**Search String PubMed (615)**

#1. ("exercise"[MH] OR "exercise"[TW] OR "exercises"[TW] OR "active living"[TW] OR "active transport"[TW] OR "activities, physical"[TW] OR "activity, physical"[TW] OR "physical activities"[TW] OR "physical activity"[TW] OR "exercise therapy"[MH] OR "physical fitness"[MH] OR fitness[TW] OR sedent*[TW] OR "sitting time"[TW] OR inactive[TW] OR inactivity[TW] OR walk*[TW])

#2. (gamification[TW] OR exergaming[TW] OR "exer-gaming"[TW])

#3. (randomized controlled trial[pt] OR controlled clinical trial[pt] OR randomized[tiab] OR clinical trials as topic[mesh:noexp] OR randomly[tiab] OR trial[ti])

#4. #1 AND #2 AND #3

#5. (game[TW] OR games[TW] OR gamified[TW] OR gaming[TW] OR gameful*[TW] OR multiplayer[TW] OR player[TW] OR players[TW] OR playing[TW])

#6. ("telemedicine"[MH] OR "telemedicine"[TW] OR "telehealth"[TW] OR e-health[TW] OR m-Health[TW] OR eHealth[TW] OR mHealth[TW] OR "mobile applications"[MH] OR "applications"[TW] OR "application"[TW] OR app[TW] OR apps[TW] OR "online"[TW] OR "mobile"[TW] OR "internet"[TW] OR "web based"[TW] OR "Smartphone"[MH] OR "phone, smart"[TW] OR "smart phones"[TW] OR "smartphones"[TW] OR "smart phone"[TW] OR "phones, smart"[TW] OR "Smartphone"[TW] OR "cell phone"[TW] OR Iphone[TW] OR android[TW] OR iOS[TW] OR "Wearable Electronic Devices"[MH] OR website[TW] OR digital*[TW] OR system*[TW] OR electronic*[TW] OR technolog*[TW] OR device[TW] OR framework*[TW] OR "social support"[Mesh:NoExp] OR "facebook"[TW] OR "networks, social"[TW])

#7. #1 AND #3 AND #5 AND #6

#8. #4 OR #7

Filters: English, from 2010-2020

**Search String EMBASE (491)**

('sport'/exp OR 'exercise'/exp OR 'physical activity' OR 'climbing'/exp OR 'walking'/exp OR 'sitting'/exp) AND ('exergame'/exp OR 'exergaming'/exp OR 'active video game'/exp OR exergam*:ti,ab,kw OR 'active video gam*':ti,ab,kw OR 'active videogam*':ti,ab,kw OR 'active computer gam*':ti,ab,kw OR 'gamification'/exp OR 'gamifi*':ti,ab,kw OR (('recreational game'/exp OR 'game-based':ti,ab,kw OR 'gam*':ti,ab,kw) AND ('telehealth'/exp OR 'mobile application'/exp OR 'smartphone'/exp OR 'wearable computer'/exp OR 'social network'/exp OR 'virtual reality'/exp))) AND ('clinical trial'/de OR 'controlled clinical trial'/de OR 'randomized controlled trial'/de OR 'randomized controlled trial topic'/de) AND [embase]/lim NOT ([embase]/lim AND [medline]/lim)

**Search String CENTRAL (1602)**

#1. ("exercise":ti,ab,kw OR "exercises":ti,ab,kw OR "active living":ti,ab,kw OR "active transport":ti,ab,kw OR "activities, physical":ti,ab,kw OR "activity, physical":ti,ab,kw OR "physical activities":ti,ab,kw OR "physical activity":ti,ab,kw OR fitness:ti,ab,kw OR sedent*:ti,ab,kw OR “sitting time”:ti,ab,kw OR inactive:ti,ab,kw OR inactivity:ti,ab,kw OR walk*:ti,ab,kw )

#2. (gamification:ti,ab,kw OR exergaming:ti,ab,kw OR “exer-gaming”:ti,ab,kw)

#3. #1 AND #2

#4. (game:ti,ab,kw OR games:ti,ab,kw OR gamified:ti,ab,kw OR gaming:ti,ab,kw OR gameful*:ti,ab,kw OR multiplayer:ti,ab,kw OR player:ti,ab,kw OR players:ti,ab,kw OR playing:ti,ab,kw)

#5. ("telemedicine":ti,ab,kw OR "telehealth":ti,ab,kw OR "e-health":ti,ab,kw OR "m-Health":ti,ab,kw OR "eHealth":ti,ab,kw OR "mHealth":ti,ab,kw OR "applications":ti,ab,kw OR "application":ti,ab,kw OR app:ti,ab,kw OR apps:ti,ab,kw OR "online":ti,ab,kw OR "mobile":ti,ab,kw OR "internet":ti,ab,kw OR “web based”:ti,ab,kw OR "phone, smart":ti,ab,kw OR "smart phones":ti,ab,kw OR "smartphones":ti,ab,kw OR "smart phone":ti,ab,kw OR "phones, smart":ti,ab,kw OR "Smartphone":ti,ab,kw OR “cell phone”:ti,ab,kw OR Iphone:ti,ab,kw OR android:ti,ab,kw OR iOS:ti,ab,kw OR website:ti,ab,kw OR digital*:ti,ab,kw OR system*:ti,ab,kw OR electronic*:ti,ab,kw OR technolog*:ti,ab,kw OR device:ti,ab,kw OR framework*:ti,ab,kw OR "facebook":ti,ab,kw OR "networks, social":ti,ab,kw OR "social network":ti,ab,kw OR "network, social":ti,ab,kw OR "social support":ti,ab,kw OR "support, social":ti,ab,kw OR "social networking":ti,ab,kw OR virtual*:ti,ab,kw)

#6. #1 AND #4 AND #5

#7. #3 OR #6

Limit: Publication Year from 2010 to 2020 in Trials.

**Search String Scopus (306)**

( ( TITLE ( "physical activity" OR sport OR exercise? OR walking OR fitness OR "active living" OR "active transport" OR sedent* OR "sitting time" OR inactiv* OR ( physical AND near/1 AND activit* ) ) ) AND ( TITLE-ABS-KEY ( "randomi?ed controlled trial" OR "randomi?ed clinical trial" OR "controlled clinical trial" OR randomi?ed OR rct ) ) AND ( TITLE-ABS-KEY ( gamifi* OR exergam* OR "exer-gam*" ) ) ) OR ( ( TITLE ( "physical activity" OR sport OR exercise? OR walking OR fitness OR "active living" OR "active transport" OR sedent* OR "sitting time" OR inactiv* OR ( physical AND near/1 AND activit* ) ) ) AND ( TITLE-ABS-KEY ( "randomi?ed controlled trial" OR "randomi?ed clinical trial" OR "controlled clinical trial" OR randomi?ed OR rct ) ) AND ( TITLE-ABS-KEY ( game? OR multiplayer OR player? OR playing OR play ) ) AND ( TITLE-ABS-KEY ( telemedicine OR telehealth OR "e-health" OR "m-Health" OR ehealth OR "mHealth" OR application? OR app? OR online OR mobile OR internet OR "web based" OR "phone, smart" OR "smart phones" OR "smartphones" OR "smart phone" OR "phones, smart" OR "Smartphone" OR "cell phone" OR iphone OR android OR "iOS" OR website OR digital* OR system* OR electronic* OR technolog* OR device OR framework* OR "facebook" OR "networks, social" OR "social network" OR "network, social" OR "social support" OR "support, social" OR "social networking" OR virtual* ) ) ) AND ( LIMIT-TO

( PUBYEAR , 2020 ) OR LIMIT-TO ( PUBYEAR , 2019 ) OR LIMIT-TO ( PUBYEAR , 2018 ) OR LIMIT-TO ( PUBYEAR , 2017 ) OR LIMIT-TO ( PUBYEAR , 2016 ) OR LIMIT-TO ( PUBYEAR , 2015 ) OR LIMIT-TO ( PUBYEAR , 2014 ) OR LIMIT-TO ( PUBYEAR , 2013 ) OR LIMIT-TO ( PUBYEAR , 2012 ) OR LIMIT-TO ( PUBYEAR , 2011 ) OR LIMIT-TO ( PUBYEAR , 2010 ) ) AND ( LIMIT-TO ( LANGUAGE , "English" ) )

**Search String Web of Science (89)**

#1. TI = ("physical activity" OR sport OR exercise? OR walking OR fitness OR "active living" OR "active transport" OR sedent* OR "sitting time" OR inactive OR inactivity OR (physical NEAR/1 activit*))

#2. TI = (gamifi* OR exergam* OR exer-gam*)

#3. TS= (“clinical trial*” OR "controlled trial*" OR "follow-up stud*" OR "prospective stud*" OR "random*" OR "single blind*" OR "double blind*" OR rct)

#4. #1 AND #2 AND #3

#5. TS= (telemedicine OR telehealth OR e-health OR m-Health OR eHealth OR mHealth OR application? OR app? OR online OR mobile OR internet OR “web based" OR "phone, smart" OR "smart phones" OR "smartphones" OR "smart phone" OR "phones, smart" OR "Smartphone" OR "cell phone" OR Iphone OR android OR "iOS" OR website OR digital* OR system* OR electronic* OR technolog* OR device OR framework* OR facebook OR "networks, social" OR "social network" OR "network, social" OR "social support" OR "support, social" OR "social networking" OR virtual*)

#6. TI = (game? OR multiplayer OR player? OR playing OR play)

#7. #1 AND #3 AND #5 AND #6

#8. #4 OR #7
