# Supplementary material for: Evaluating the Effectiveness of Gamification on Physical Activity: Systematic Review and Meta-analysis of Randomized Controlled Trials
Source: J Med Internet Res. 2022 Jan 4;24(1):e26779. doi: 10.2196/26779 (PMC8767479; doi:10.2196/26779)
Supplement: Multimedia Appendix 2 [file jmir_v24i1e26779_app2.pdf]

Risk of bias summary for studies included in the meta-analysis

Judgement: X High; - Unclear; + Low

Study	Random sequence generation	Allocation concealment	Blinding of outcome assessment	Incomplete outcome data	Selective reporting	Other bias	Overall
Corepal et al., 2019	+	+	+	X	+	X	X
Dadaczynski et al., 2017	+	-	X	X	+	+	X
Direito et al., 2015	+	+	+	+	+	+	+
Edney et al., 2020	+	+	+	+	+	+	+
Garde et al., 2016	+	+	+	X	-	+	X
Gremaud et al., 2018	+	+	+	+	+	+	+
Höchsmann et al., 2019	+	+	+	+	+	+	+
Kurtzman et al., 2018	+	+	+	+	+	+	+
Leinonen et al., 2017	+	+	+	X	+	+	X
Maher et al., 2015	+	+	X	+	+	+	X
Nishiwaki et al., 2014	-	-	+	+	-	X	X
Patel et al., 2017	+	+	+	+	+	+	+
Patel et al., 2019	+	+	+	+	+	+	+
Paul et al., 2016	X	X	+	+	-	+	X
Thorsteinsen et al., 2014	+	-	X	X	-	+	X
Zuckerman & Gal-Oz, 2014	-	-	+	+	-	+	+
