# Supplementary material for: Evaluating the Effectiveness of Gamification on Physical Activity: Systematic Review and Meta-analysis of Randomized Controlled Trials
Source: J Med Internet Res. 2022 Jan 4;24(1):e26779. doi: 10.2196/26779 (PMC8767479; doi:10.2196/26779)

Risk of bias graph: review authors' judgments about each risk of bias item presented as percentages across all included studies

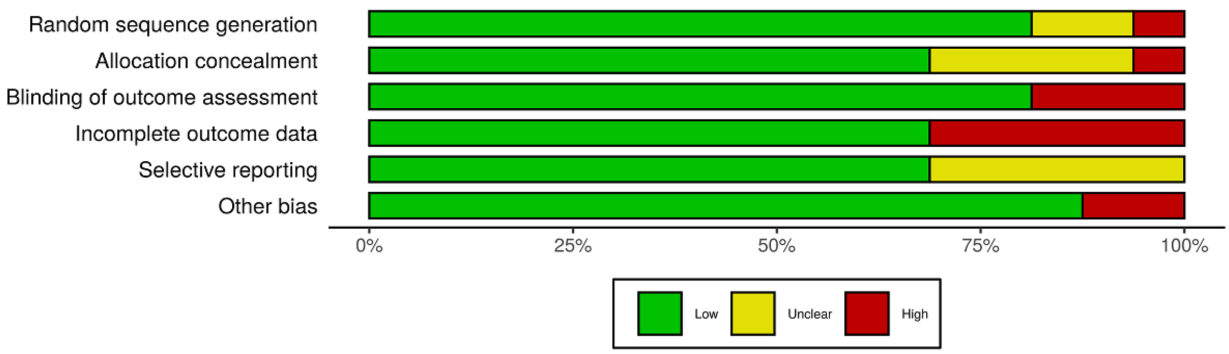

Supplement: Multimedia Appendix 3 [file jmir_v24i1e26779_app3.pdf]
